# Particle jet impact deep-rock in rotary drilling: Failure process and lab experiment

**DOI:** 10.1371/journal.pone.0250588

**Published:** 2021-04-28

**Authors:** Tiancheng Fang, Fushen Ren, Baojin Wang, Jianxun Cheng, Hanxu Liu

**Affiliations:** Northeast Petroleum University, Daqing, Heilongjiang, China; University of Science and Technology Beijing, CHINA

## Abstract

Aimed at the technical problems of low drilling speed and difficult rock-breaking in deep-well and hard rock-stratum, particle waterjet coupled impact rock-breaking technology in rotary drilling is put forward in this paper. Firstly, the working principle of particle jet impact rock-breaking in rotary drilling was introduced, and the acceleration model of particle jet and the damage model of rock were established. The acceleration mechanism of particles and dynamic damage evolution process of rock under particle jet were studied, which showed that the broken pit and rock damage would increase with time gone on, and damage evolution of rock presented the radial expansion. Then, experimental device of particle jet coupled impact rock-breaking in rotary state was developed, and the effect of jet parameters on penetration depth and failure volume was analyzed with comparison of la experiment and numerical simulation. The results showed that drilling speed with particle jet impact is twice that of conventional drilling, and combination nozzles layout of impact angle with 8°and 20° can achieve rock-drilled rapidly, which also demonstrated the correctness of simulation method. The device development and the rock-breaking results analysis would be of great value for engineering application.

## Introduction

**Fig 1:**
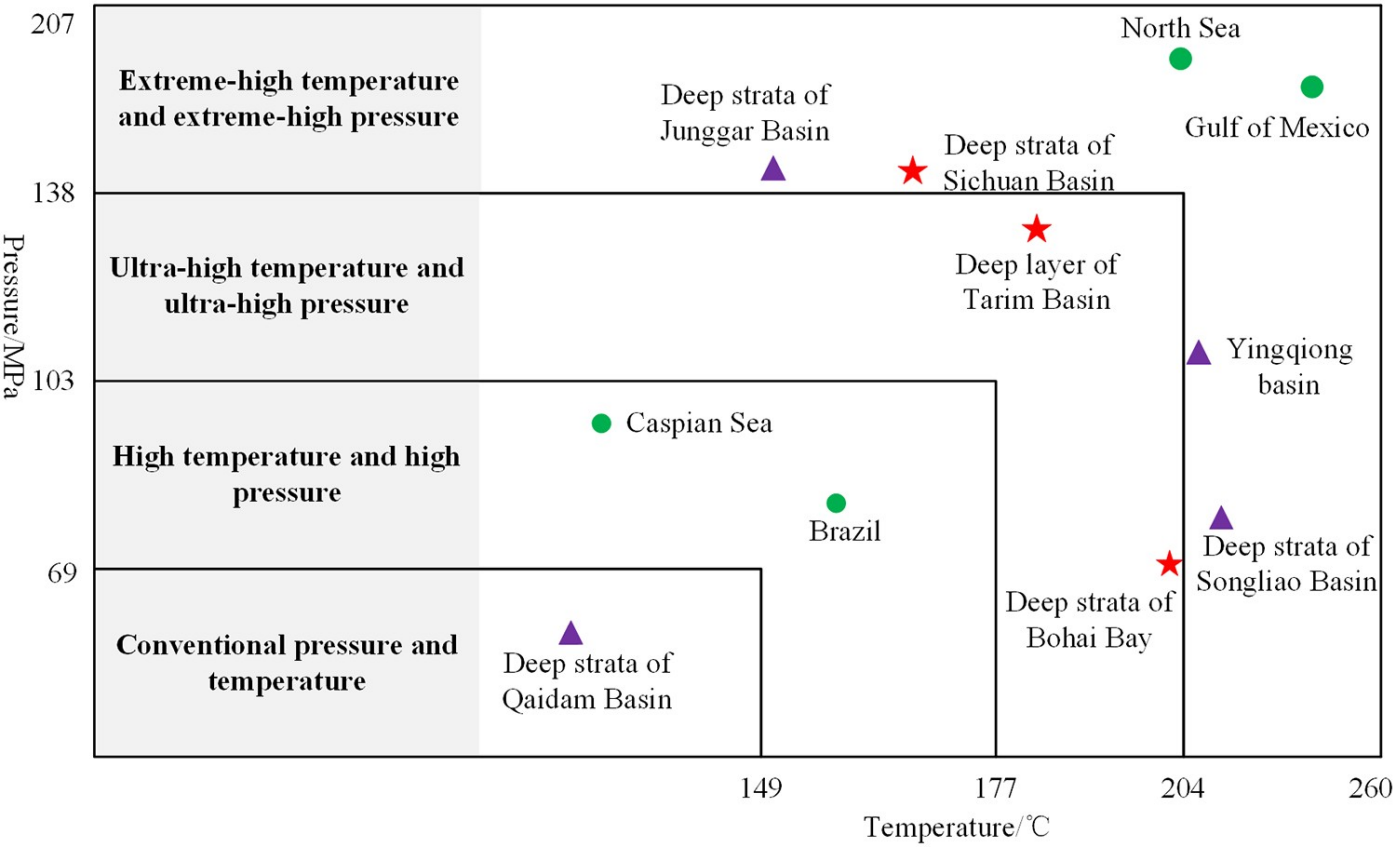
Pressure and temperature environment of some deep-well reservoirs at home and abroad.

With depletion of shallow oil resources and complexity of exploration geology, the drilling depth is greatly increasing, and the drilling conditions are becoming more complex, such as high temperature, high pressure and high hardness, and Fig 1 is shown the temperature and pressure distributions of some deep-well of oil and gas reservoirs at home and abroad [1]. At present, the rock-breaking technology of abrasion resistant formation in deep-well and ultra-deep-well would become an urgent technical research of China [2]. In drilling process of deep-well hard formation, the deeper the drilling depth, the more serious the bit wear, and the higher the drilling cost. Therefore, drilling speed and rock-breaking efficiency in abrasion resistant formation become the key technologies to restrict rapidly development of deep-well drilling [3]. In recent years, non-contact rock-breaking technology has developed rapidly and is becoming an important approach to achieve rapid drilling in deep-well.

Particle jet coupled impact rock-breaking technology is an efficient non-contact rock-breaking method, which mainly uses subsonic spherical particles to break rock, and high-velocity waterjet is used to act to remove debris and impact rock auxiliary. Then, hard rock is formed to well-bore with bit teeth cutting and grinding [4, 5]. Therefore, the rock-breaking mechanism is that fracture zones and damage areas of hard rock are impacted by particles, then the impact of waterjet can aggravate rock damage, and finally the rock released confining pressure is broken with action of bit rotary cutting. Compared with traditional drilling, the efficiency of particle waterjet impact drilling can be increased by about twice times, which is suitable for hard formation section of ultra-deep wells. However, the rock-breaking technology has complex ground equipment such as particle injection equipment and recovery equipment, and has certain challenges to the pressure and service life of pipelines. The technology has great development potential and gradually applied in fields of oil drilling, coal exploitation and underground engineering [6–8].

Recently, study on particle jet coupled impact rock-breaking technology was mainly carried by some research scholars with field application and laboratory test. PDTI, the particle impact drilling company in America, has conducted many laboratory simulation experiments and improved field tests, which proves the feasibility and advantage of drilling method [9]. Cui et al. studied the effect of jet, hydraulic and particle size on rock fragmentation through laboratory simulation test [10]. And particle classification device, and particle injection system were studied and the influence of different concentrations of particles on rock-breaking effect analyzed [11, 12]. The indoor experiment and field experiment of particle jet impact rock-breaking were carried out, which verified the feasibility of particle impact drilling technology [13, 14]. Kovalyov et al. used the method of high-speed camera to capture the movement state of metal particles in the flow channel, and proposed a structural scheme of metal particles recycling in the drill [15]. Besides, the mechanism of rock failure was studied, and the matching relationship between particle size, impact velocity, fluid velocity and rock-breaking volume is proposed [16, 17]. At present, these researches are mainly focused on fixed-point impact with single nozzle, and the researches on particle impact rock fracture in rotary drilling and cutting of bit are relatively lacking.

Based on the principle of particle jet coupled impact rock-breaking technology in rotary drilling, the numerical model of rock-breaking is established and the laboratory experimental device is developed respectively. The change of penetration depth and failure volume under different jet parameters are studied with verification of simulation and experiment. By compared with traditional drilling method, the advantages and engineering application significance of particle jet coupled impact rock-breaking technology are further clarified.

## Working principle and rock-breaking mechanism

### Working principle of rock-breaking technology

**Fig 2:**
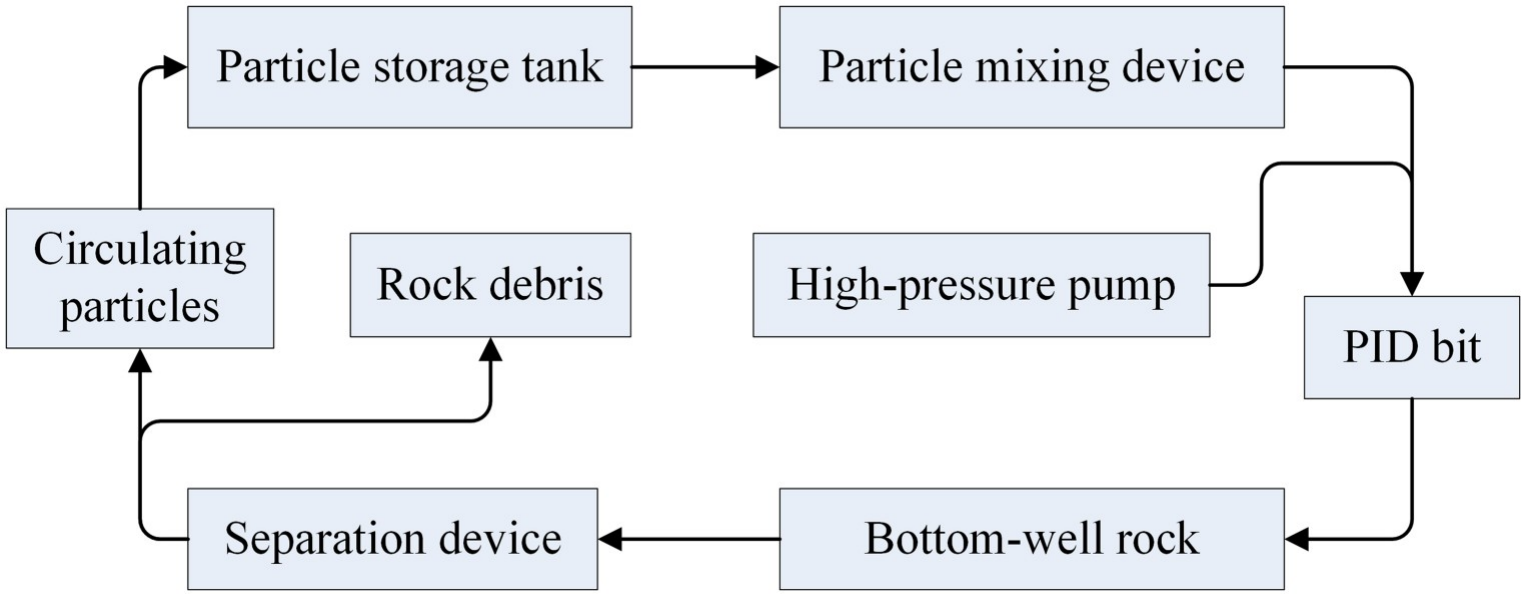
The rock-breaking process of particle jet coupled impact.

Particle jet coupled impact drilling technology was proposed based on projectile impact [9, 18], and its working principle is to add a certain proportion of steel particles into the drilling fluid, and then the two-phase fluid would accelerate through the special nozzle to form subsonic particle waterjet. The high-velocity particle waterjet would firstly impact and break rock to form the pressure-relief pit on rock surface, which would release compaction stress in deep-well and the crushing strength of damaged rock would reduce greatly [5, 16]. And then, the damaged rock would be formed to drilling hole under the action of rotary cutting and grinding by bit teeth. Meanwhile, the cutting debris and steel particles could be removed by drilling fluid. The rock-breaking process of particle jet coupled impact is shown in Fig 2.

Compared with conventional drilling technology, particle jet coupled impact rock-breaking technology mainly relies on high-frequency and high-velocity steel particles to break rock at the bottom of deep-well, which has the advantages of high rock-breaking efficiency, fast drilling speed and small deviation.

### Modeling and simulation

#### Modeling and analysis of jet acceleration impact

In particle jet coupled impact drilling technology, two phase flow would mainly be accelerated through nozzle. When waterjet carries steel particles from high-pressure pipeline into nozzle, the cross-sectional area of accelerating nozzle would change, so the particle jet velocity would also change [19, 20]. And the acceleration of nozzle can be divided into contraction acceleration and cylinder acceleration.

**Fig 3:**
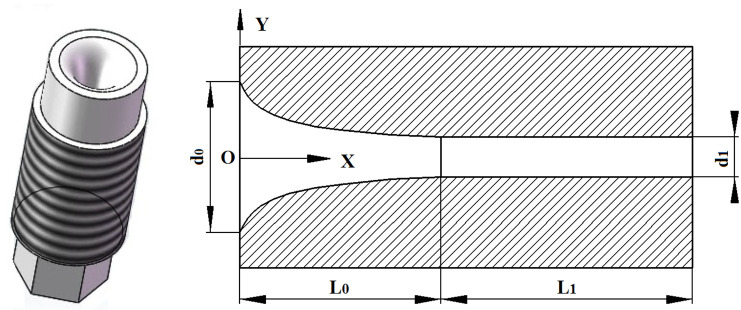
The nozzle section and coordinate system.

In the modeling process of particle jet acceleration, it is assumed that steel particles and water could be uniformly mixed. The coordinate system is established in the center of the nozzle inlet. The radial direction of nozzle is Y-axis, and the depth direction of nozzle is X-axis, as shown in Fig 3. Analyzed contraction section of accelerating nozzle, the acceleration model of particle and jet is obtained by the Bernoulli equation and second Newton’s law:

$$v_{f}\pi y^{2}=\frac{1}{4}\pi d_{0}{}^{2}v_{f0}$$ (1)

$$v_{p}\frac{dv_{p}}{dx}=\frac{3C_{D}\rho_{f}}{D_{P}\left(4\rho_{p}+2\rho_{f}\right)}{\left(v_{f}-v_{p}\right)}^{2}+\frac{3\rho_{f}}{2\rho_{p}+\rho_{f}}\frac{v_{f}dv_{f}}{dx}$$ (2)

Where, *v*_*f*0_ is the jet velocity at nozzle inlet; *v*_*f*_ is the velocity variable of waterjet; *C*_*D*_ is the viscosity coefficient of jet [20]; *d*_0_ is the diameter of nozzle inlet; *v*_*p*_ is particle velocity; *D*_*p*_ is particle diameter; *ρ*_*p*_ and *ρ*_*f*_ represent the density of particle and jet respectively.

Then, analyzed cylinder section of accelerating nozzle, the acceleration model of particle waterjet is:

$$v_{f}d_{1}{}^{2}=v_{f0}d_{0}{}^{2}$$ (3)

$$v_{p}dv_{p}=\frac{3C_{D}\rho_{f}}{D_{P}\left(4\rho_{p}+2\rho_{f}\right)}{\left(v_{f}-v_{p}\right)}^{2}dx$$ (4)

Where, *d*_1_ is the diameter of nozzle outlet.

The flow state of the fluid is judged by the Reynolds coefficient. Because the two-phase flow of particle and water has high speed and great change in accelerating process, therefore, in the calculation of the flow field, the flow state of jet is turbulent, and its Reynolds number and turbulence intensity can be obtained [21]:

$$\text{Re}=\frac{\rho_{f}v_{f}d_{1}}{C_{D}},\;I=0.16{\left(\frac{\rho_{f}v_{f}d_{1}}{C_{D}}\right)}^{-0.125}$$ (5)

Where, Re is the Reynolds of fluid; *I* is the turbulence intensity. Generally speaking, when Reynolds number Re < 2300, the flow of fluid is laminar stage, and when Reynolds number Re > 40000, the flow of fluid is turbulent state. When the flow of fluid is transition state, the Reynolds number meets the requirement of Re = 2300 ~ 4000.

The RNG *k* − *ε* equation is used to describe the turbulent flow of particle jet when the computational fluid dynamics method is used to calculate the fluid flow:

$$\frac{\partial \left(\rho_{f,p}k\right)}{\partial t}+\frac{\partial \left(\rho_{f,p}kv\right)}{\partial x_{i}}=\frac{\partial }{\partial x_{j}}\left[\left(C_{D}+\frac{C_{D}}{\sigma_{k}}\right)\frac{\partial k}{\partial x_{j}}\right]+G_{k}+G_{b}-k\varepsilon -Y_{M}$$ (6)

$$\frac{\partial \left(\rho_{f,p}\varepsilon \right)}{\partial t}+\frac{\partial \left(\rho_{f,p}\varepsilon v\right)}{\partial x_{i}}=\frac{\partial }{\partial x_{j}}\left[\left(C_{D}+\frac{C_{D}}{\sigma_{\varepsilon }}\right)\frac{\partial \varepsilon }{\partial x_{j}}\right]+G_{1\varepsilon }\frac{\varepsilon }{k}\left(G_{k}+C_{3\varepsilon }G_{b}\right)-G_{2\varepsilon }\rho_{f,p}\frac{\varepsilon^{2}}{k}$$ (7)

Where, *k* is the turbulent kinetic energy; *t* is the time; *ε* is the dissipation rate of turbulence; *G*_*k*_ and *G*_*b*_ are the kinetic energy due to velocity gradient and buoyancy; *x*_*i*_ and *x*_*j*_ are the displacement component; *Y*_*M*_ is the compressibility of turbulent fluid, and its value is 0; *G*_1*ε*_, *G*_2*ε*_ and *G*_3*ε*_ are the turbulent constant; *σ*_*ε*_ and *σ*_*k*_ are the Prandtl constant [22].

For the acceleration model of particle jet, calculation solution and simulation solution are obtained by MATLAB and FLUENT respectively, and the velocity obtained is compared and analyzed. In the process of simulation, the RNG *k* − *ε* turbulence equation is used for flow field, and the SIMPLE algorithm is used for discrete equation of computational fluid model. In calculation and simulation process, the parameters of particle and waterjet are shown in Table 1.

**Table 1:** The parameters of particle and waterjet.

jet density *ρ*_*f*_	Particle density *ρ*_*p*_	jet velocity at inlet *v*_*f*0_	Particle diameter *D*_*P*_	Viscosity coefficient *C*_*D*_
1100 kg/m^3^	7800 kg/m^3^	5~20 m/s	1.0 mm	17.2 μPa·s
Diameter of inlet *d*_0_	Diameter of outlet *d*_1_	Contraction length *L*_0_	Cylinder length *L*_1_	Compressibility of fluid *Y*_*M*_
15 mm	4 mm	15 mm	40 mm	0

**Fig 4:**
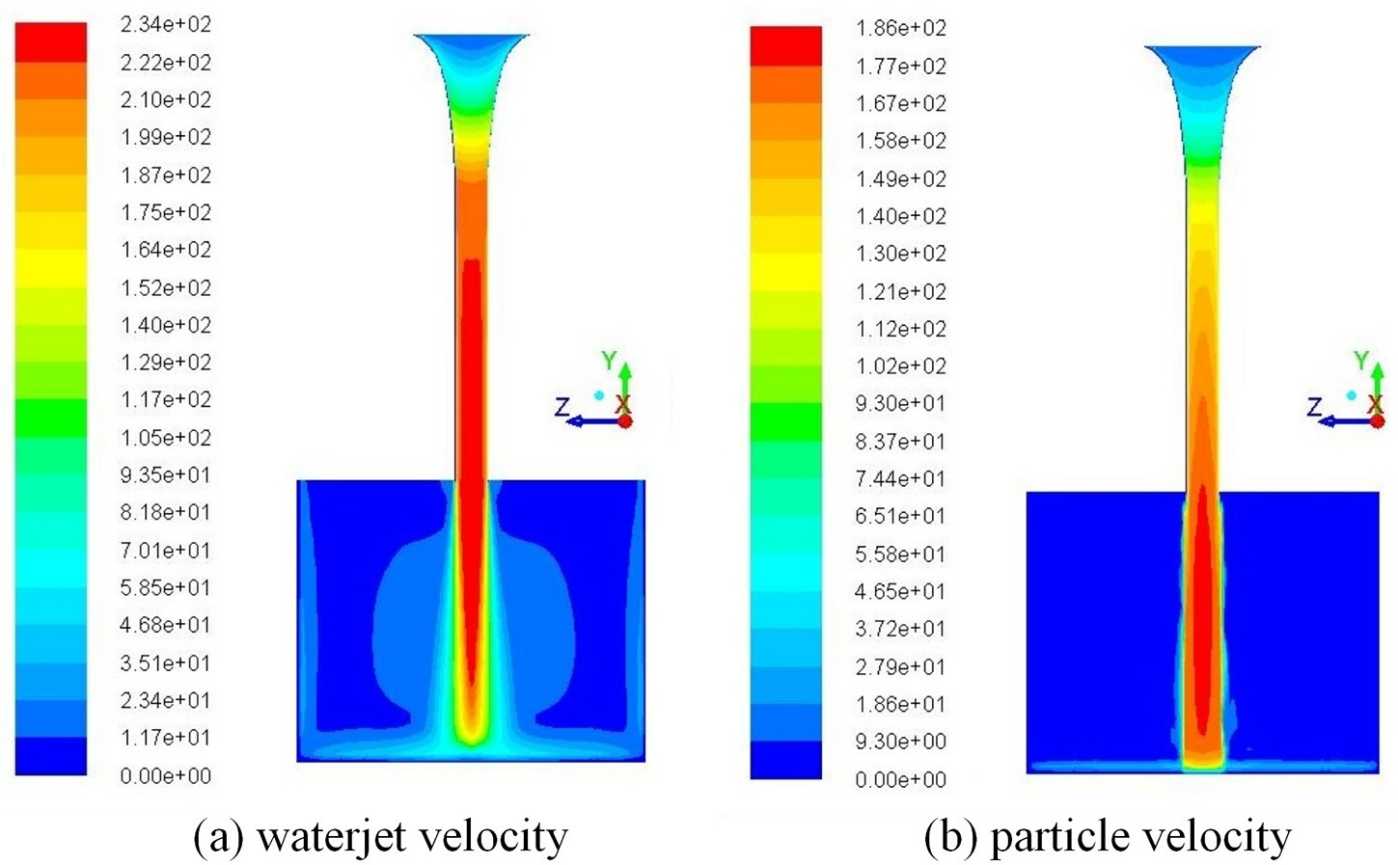
The distribution of particle velocity and waterjet velocity.

According to the established acceleration model, the velocity distribution and flow field of particle and waterjet is as shown in Fig 4. The acceleration process of waterjet is mainly concentrated in contraction section, and the velocity of waterjet in cylindrical section is basically unchanged, while the acceleration process of particles runs through the contraction and cylinder section. This is because the acceleration of particles lags behind jet, and the velocity of waterjet in cylindrical section is basically unchanged, while its velocity is obviously higher than that of particles, so particles will continue to accelerate with carried by high-speed waterjet. The jet core region would be formed outside area of nozzle, and the velocity of waterjet gradually decreases, while the velocity of particles would increase first and then decrease.

**Fig 5:**
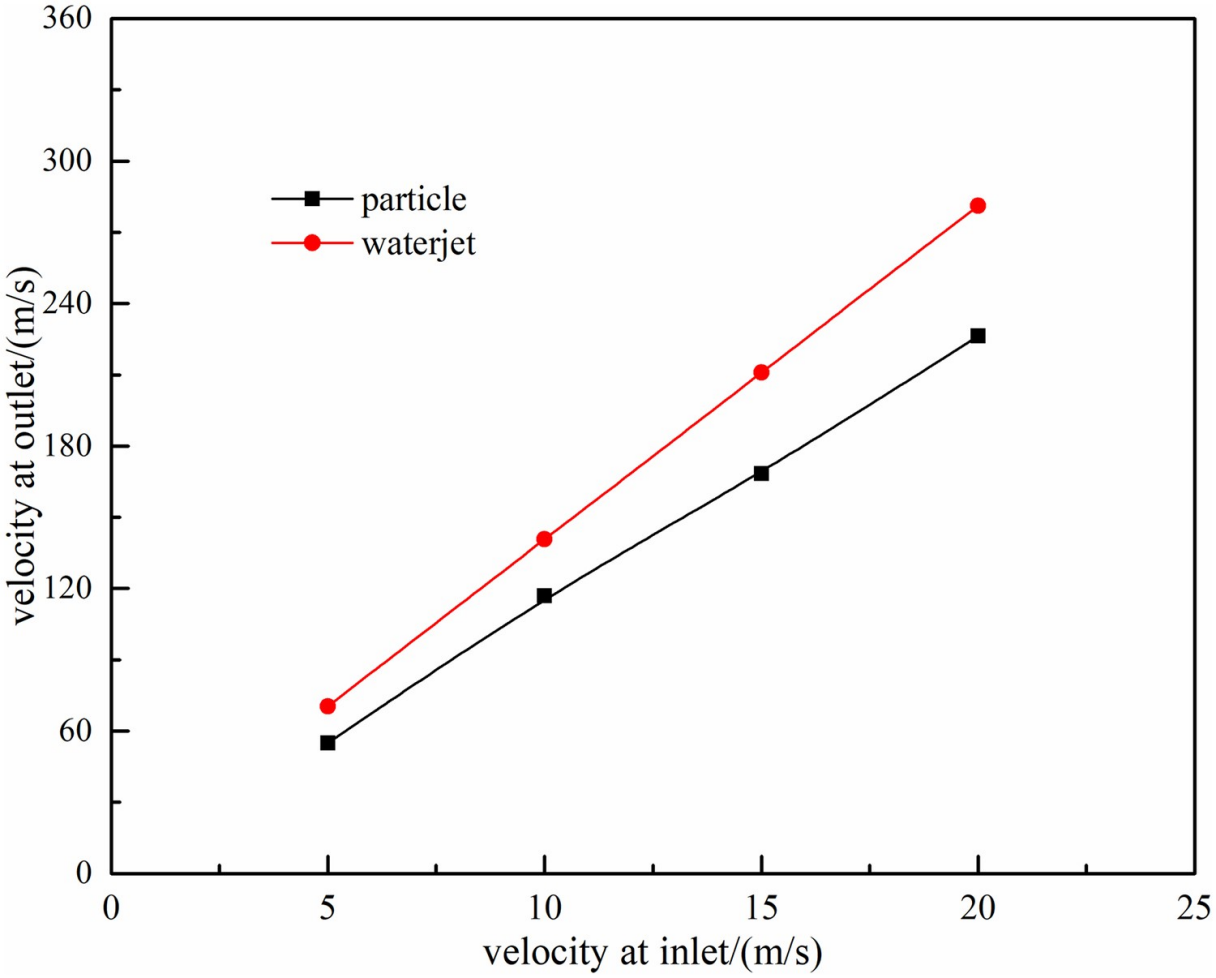
The effect of velocity change at nozzle inlet on acceleration of nozzle.

When steel particles and water jet flow through the long high-pressure pipeline to mix evenly, the velocity of particle and jet at nozzle inlet is basically the same. When the flow rate of power pump is different, the velocity at nozzle inlet will also be different. The effect of velocity change at nozzle inlet on the acceleration of nozzle is shown as Fig 5.

According to the calculation of established acceleration model, the velocity of particle and waterjet at nozzle outlet would increase linearly with that of at nozzle inlet. And for accelerating nozzle, jet velocity can be accelerated to about 13 times, and particle velocity can be accelerated to about 11 times. This model can provide support for the pump power selection of particle jet impact rock-breaking.

#### Rock damage model and mechanism analysis

When impact velocity of particle jet reaches subsonic speed, fracture and internal damage would appear in rock. The rock-breaking mechanism under particle jet coupled impact is mainly due to powerful instantaneous impact stress on rock. When high-velocity metal particles impact on rock surface, the instantaneous impact stress would produce in a very small contact area and the stress value is very large. When particle size, impact velocity and other parameters reach the certain value, the instantaneous impact stress would exceed the compressive strength of hard rock, and tensile stress and shear stress will occur around the boundary of contact area. When tensile stress or shear stress exceeds the tensile or shear strength of rock respectively, many cracks or macroscopic failure will be formed inside rock [5, 23].

At present, the failure models applied to impact and collision field mainly include Holmquist-Johnson-Cook (HJC) model, Drucker-Prager (DP) model, Riedel-Hiermaier-Thoma (RHT) model, and Johnson-Holmquist (JH-2) model [16, 24, 25]. HJC model is mainly used for brittle elastic materials such as glass and ceramics; DP model is mainly used to simulate ideal elastic-plastic models such as cohesive soil; RHT model is suitable for concrete materials; JH-2 model is expressed as the functions of pressure, damage and strain, which can realize dynamical simulation and analysis of rock fragmentation. Therefore, JH-2 model is proposed to describe the rock-breaking phenomenon and damage evolution under particle waterjet impact.

The relationship between volume strain rate and static pressure can be expressed by the state equation. With the high-speed impact of particle waterjet, the high-pressure state equation is needed. Therefore, the cubic-state equation is:

$$P_{r}=K_{1}\psi +K_{2}\psi^{2}+K_{3}\psi^{3}$$ (8)

Where, *P*_*r*_ is static pressure; *K*_1_ is volume model; *K*_2_ and *K*_3_ are constant; *ψ* is strain rate, *ψ* = *ρ*/*ρ*_*r*_ − 1; *ρ*_*r*_ is current density; *ρ*_0_ is initial density.

Introducing strain rate and damage factor to characterize the strength model of material [26], its normalized equivalent stress model can be expressed as:

$$\sigma^{*}=\sigma_{i}^{*}-D\left(\sigma_{i}^{*}-\sigma_{f}^{*}\right)$$ (9)

Where,$\sigma_{i}^{*}$ is equivalent stress of undamaged material, and $\sigma_{i}^{*}=\sigma_{i}/\sigma_{Hel}$; *σ*_*Hel*_ is effective stress of elastic limit; $\sigma_{f}^{*}$ is equivalent stress at completely broken stage; *D* is damage factor.

JH-2 model is adopted for rock material in simulation process, which can well simulate the broken and damage accumulation of rock under coupled impact of particle waterjet [27, 28]. The cumulative expression of JH-2 model is as follows:

$$D=\sum \frac{\Delta \varepsilon_{p}}{D_{1}{\left(P^{*}+T^{*}\right)}^{D_{2}}}$$ (10)

Where, Δ*ε*_*p*_ is effective plastic strain in an integral period;$\varepsilon_{p}^{f}$ is elastic strain of equivalent fracture; *D*_1_ and *D*_2_ is damage constant.

To determine the parameters of rock, the shear modulus *G* and bulk modulus *K*_1_ are firstly determined through the tensile-compressive test and these basic physical parameters (such as, density *ρ*_*r*_, Poisson’s ratio *μ* and elastic modulus *E*) [5, 29]:

$$G=E/2\left(1+\mu \right)$$ (11)

$$K_{1}=E/3\left(1-2\mu \right)$$ (12)

Meanwhile, for the effective stress of elastic limit *σ*_*Hel*_:

$$\sigma_{Hel}=F_{c}\left(1-\mu \right)/\left(1-2\mu \right)$$ (13)

Where, *F*_*c*_ is compressive strength, and it has been measured by rock compression test [16, 30].

The JH-2 model is used to simulate rock failure effect, and it is also coupled with the fracture softening model to simulate plastic compression and shear failure effects, and also crack propagation inside of rock target [27, 31]. To sum up, the model parameters in the simulation process of rock damage and failure under particle jet impact are summarized in Table 2.

**Table 2:** The model parameters of rock damage simulation.

Rock density *ρ*_*r*_	Bulk modulus *K*_1_	Shear modulus *G*	Poisson’s ratio *μ*
2600 kg/m^3^	25.6 GPa	20.4 GPa	0.30
Damage constant *D*_1_	Damage constant *D*_*2*_	Compressive strength *F*_*c*_	Effective stress of elastic limit *σ*_*Hel*_
0.05	0.95	55 MPa	4.52 GPa

**Fig 6:**
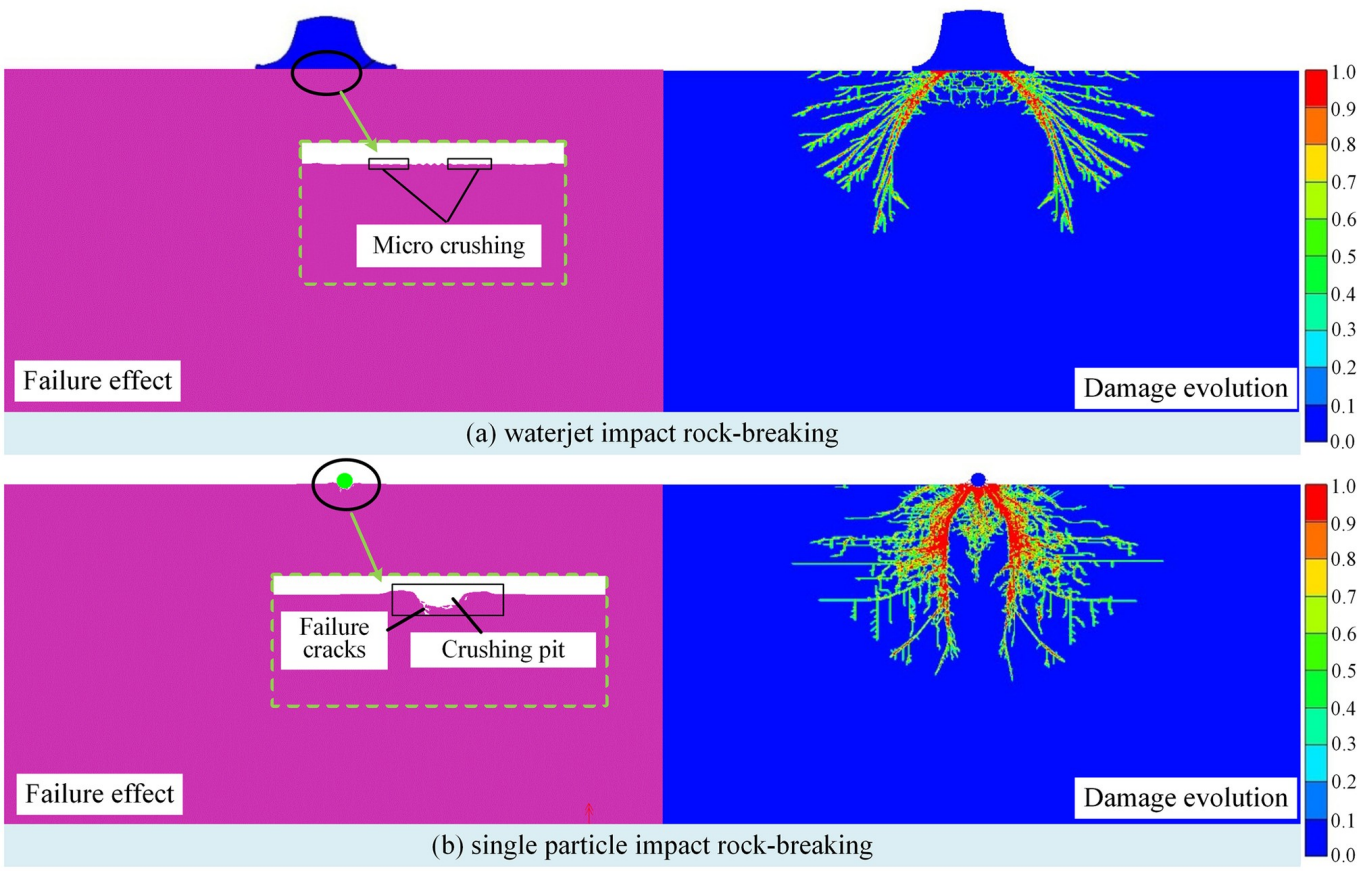
The rock-breaking effect under waterjet impact and particle impact.

Based on the above modeling method, the simulation model and rock-breaking effect under waterjet impact and single particle impact are established and simulated dynamically, as shown in Fig 6. For waterjet impact, only a small amount of fracture appears on rock surface at the edge of jet impact area, while the particle impact not only causes plastic damage at the impact point, but also causes some cracks inside of rock. Therefore, according to simulation analysis, the rock-breaking effect is mainly impacted by particles, and waterjet can aggravate damage with the auxiliary role in rock-breaking process.

**Fig 7:**
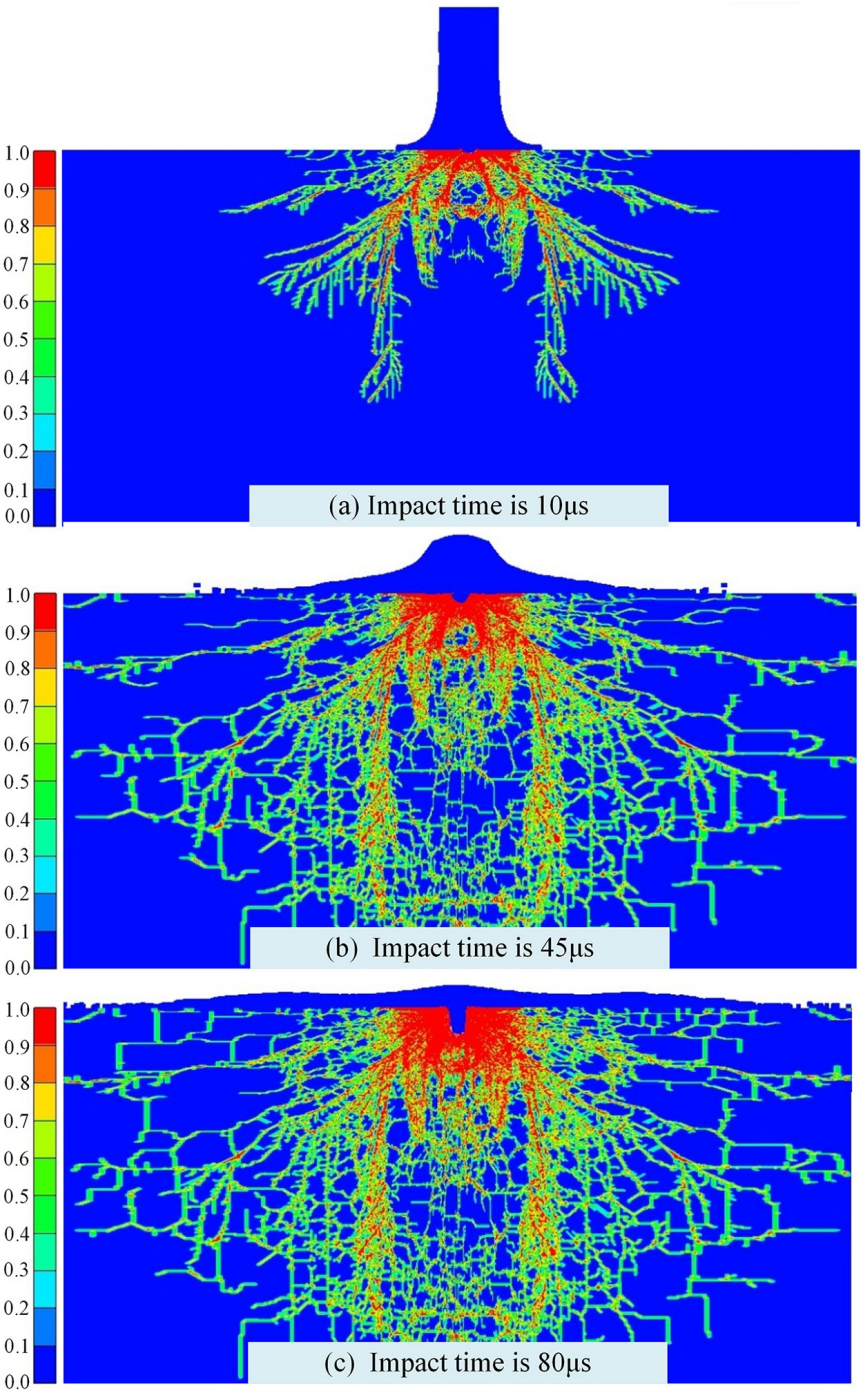
Damage evolution and broken effect of particle jet coupled impact rock.

Under multi-particles and jet impact, the dynamic simulation of rock-breaking is carried out, damage evolution and broken effect of rock under particle jet coupled impact is simulated. The failure effect and damage evolution status under multiple particles and waterjet impact on the same place (impact velocity is 200 m/s, particle size is 1 mm) is shown in Fig 7.

According to the figure of rock failure effect, rock-breaking depth increases with the impact times, but is not only simply superposition. The main reason is that it will be formed the small damage area under the first impact, and also produced pits and many dominant and hidden cracks. When the particle impact rock again, it is easier to realize the larger fracture area and be formed the deeper damage. Besides, the impact of jet can also remove debris and cause crack propagation. Under such cyclic impact of particles and waterjet, rock-breaking depth will be accumulated and intensified. Meanwhile, from the damage evolution, with the coupled impact of particles and waterjet, longitudinal damage expansion would be appeared firstly and accompanied by miniature broken pit. As time goes on, the broken pit and longitudinal damage of rock are increasing with increase of time. The broken pit section present shape of “cylindrical” and the damage section present shape of “Radial-shaped”.

**Fig 8:**
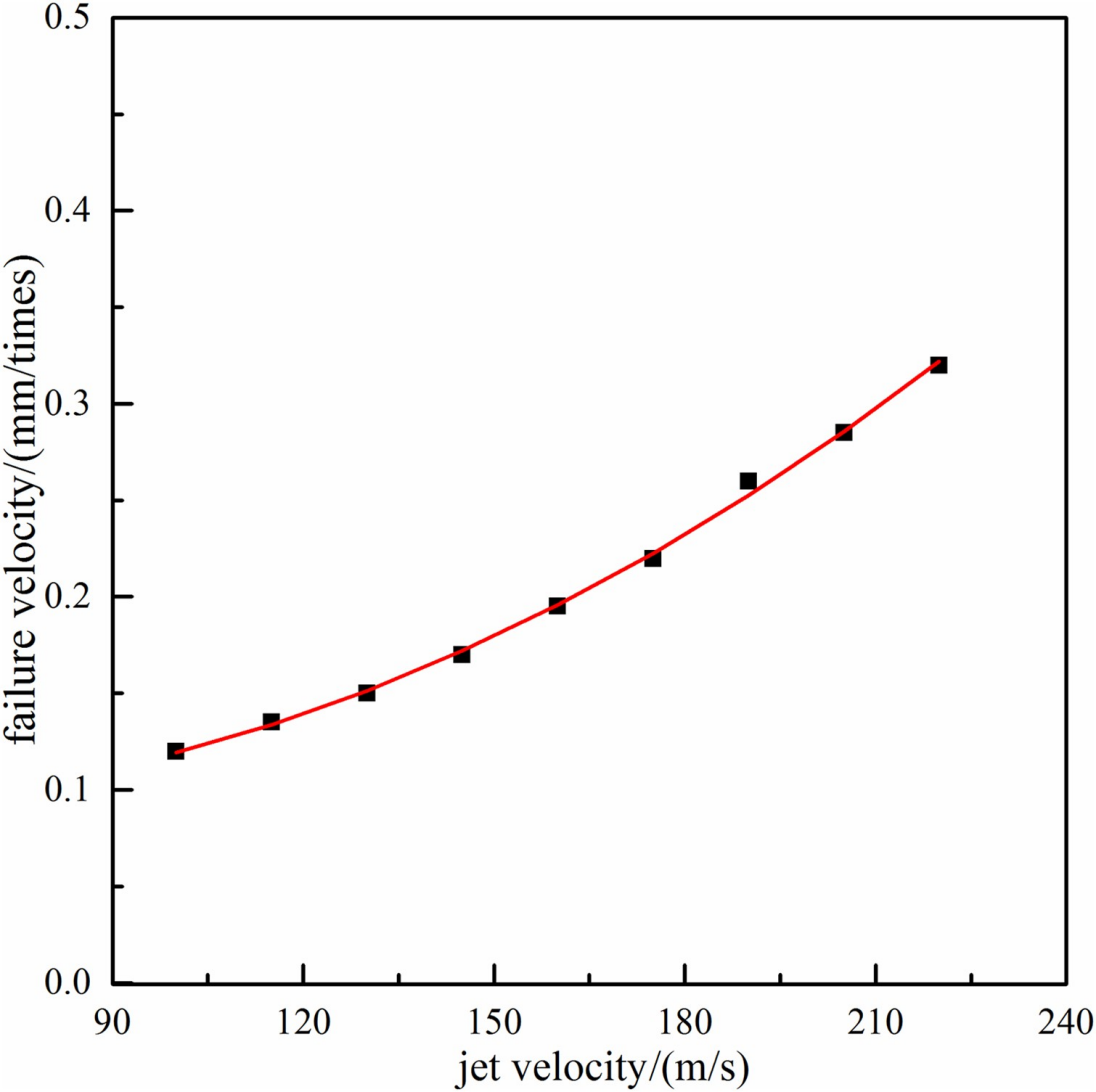
The effect law of rock-breaking velocity with jet velocity.

Based on the above dynamical simulation model, the effect law of particle jet impact rock-breaking depth under different impact velocity (jet velocity within 100m/s~220m/s) is shown in Fig 8.

With increase of jet impact velocity, the rock-breaking velocity would also increase, and presents the trend of slowly growth at first and then approximately linear growth. The main reason is that when particle diameter is fixed, the greater the jet impact velocity, the larger the particle velocity with acceleration of waterjet, and the greater the rock-breaking kinetic energy under particle jet impact. Because jet velocity is mainly determined by the pressure and flow of power pump, the higher the jet impact velocity, the higher the requirement of surface equipment. Therefore, on the premise of ensuring the safety of surface equipment, the lager impact velocity should be adopted as far as possible to improve rock-breaking efficiency and drilling speed.

According to the analysis of acceleration model and impact rock breaking model of particle waterjet, it can be seen that, in the rock-breaking process under particle jet coupled impact, the impact times per minute of steel particles can reach more than four million times, and impact velocity of particle is more than 150 m/s (jet velocity is more than 160m/s). Under such high-frequency and high-velocity impact, rock-breaking speed would be multiplied.

## Experiment results and discussion

### Overall scheme and key technology

**Fig 9:**
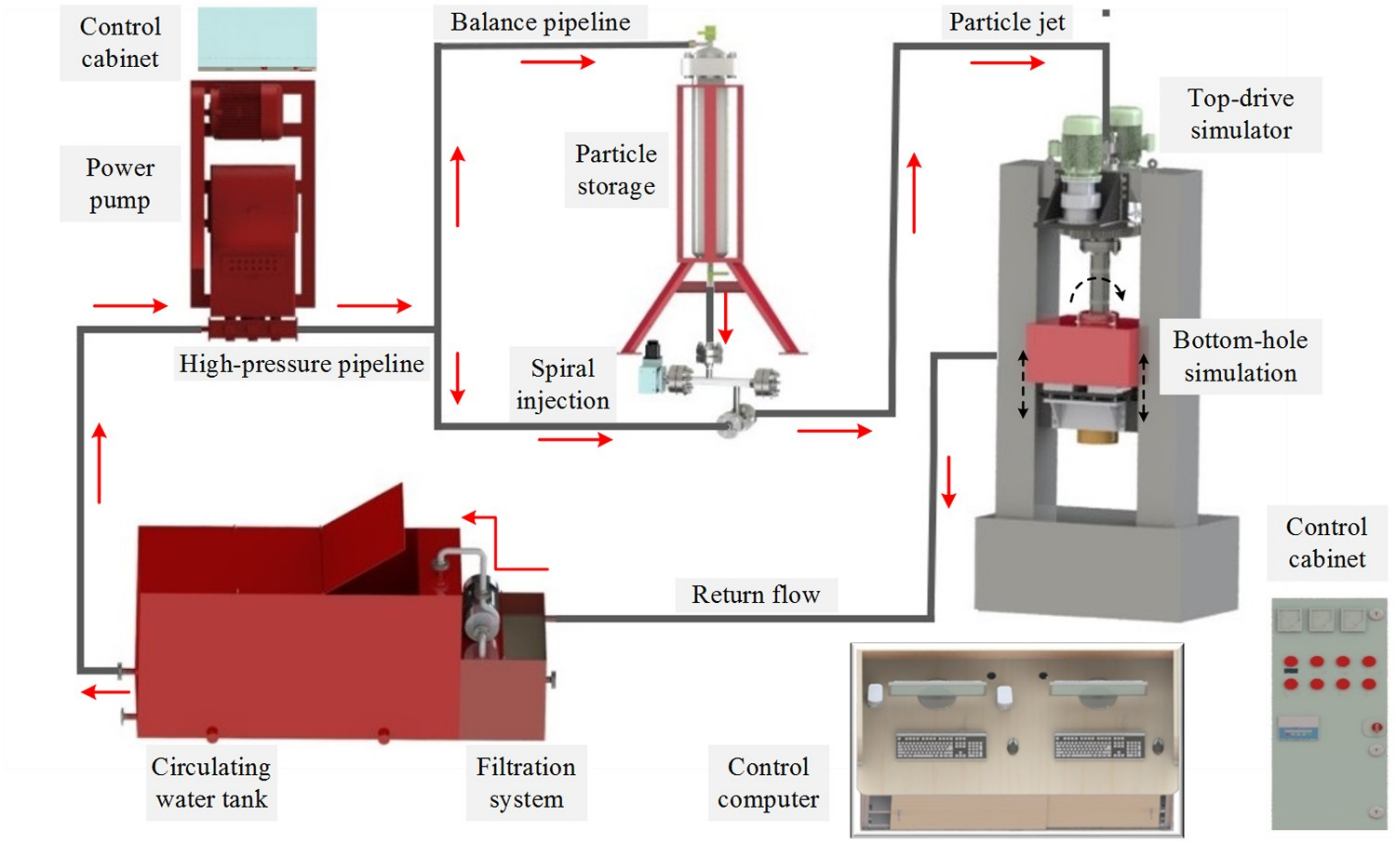
Overall design scheme of particle jet coupled impact drilling.

According to the working principle of particle jet coupled impact drilling technology in rotary drilling, the overall design scheme of laboratory experimental device is put forward, as shown in Fig 9. Laboratory experimental device of particle jet coupled impact rock-breaking mainly includes power system, particle mixing system, simulated top-drive system, simulated bottom-well system, PID (Particle Impact Drilling) bit, water circulation system, high-pressure pipeline system and control system. Among them, the key technologies include particle mixing system, PID bit, simulated top-drive system, simulation bottom-well system.

**Fig 10:**
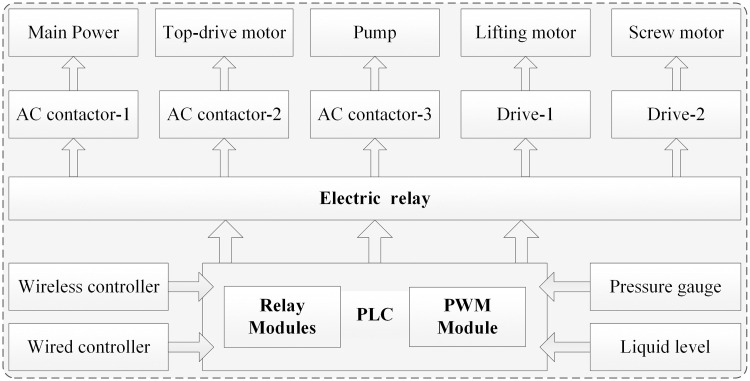
The modules diagram of control system.

The power system is mainly composed of high-pressure plunger pump (GPB-90F) and control cabinet, which could ensure hydrodynamic force supply for rock-breaking experiment. It adopts step-down starting mode and is equipped with remote control device to realize remote control. The water circulation system can not only realize the recycling of drilling fluid, but also realize the separation of metal particles and cutting debris, so as to save resources. The high-pressure pipeline system and control system could realize the sealed connection and precise control of independent key devices. PLC and its supporting modules are used to realize the monitoring of motor speed, pressure signal and position signal. The diagram of control system is shown in Fig 10.

### Device development and experimental sample

**Fig 11:**
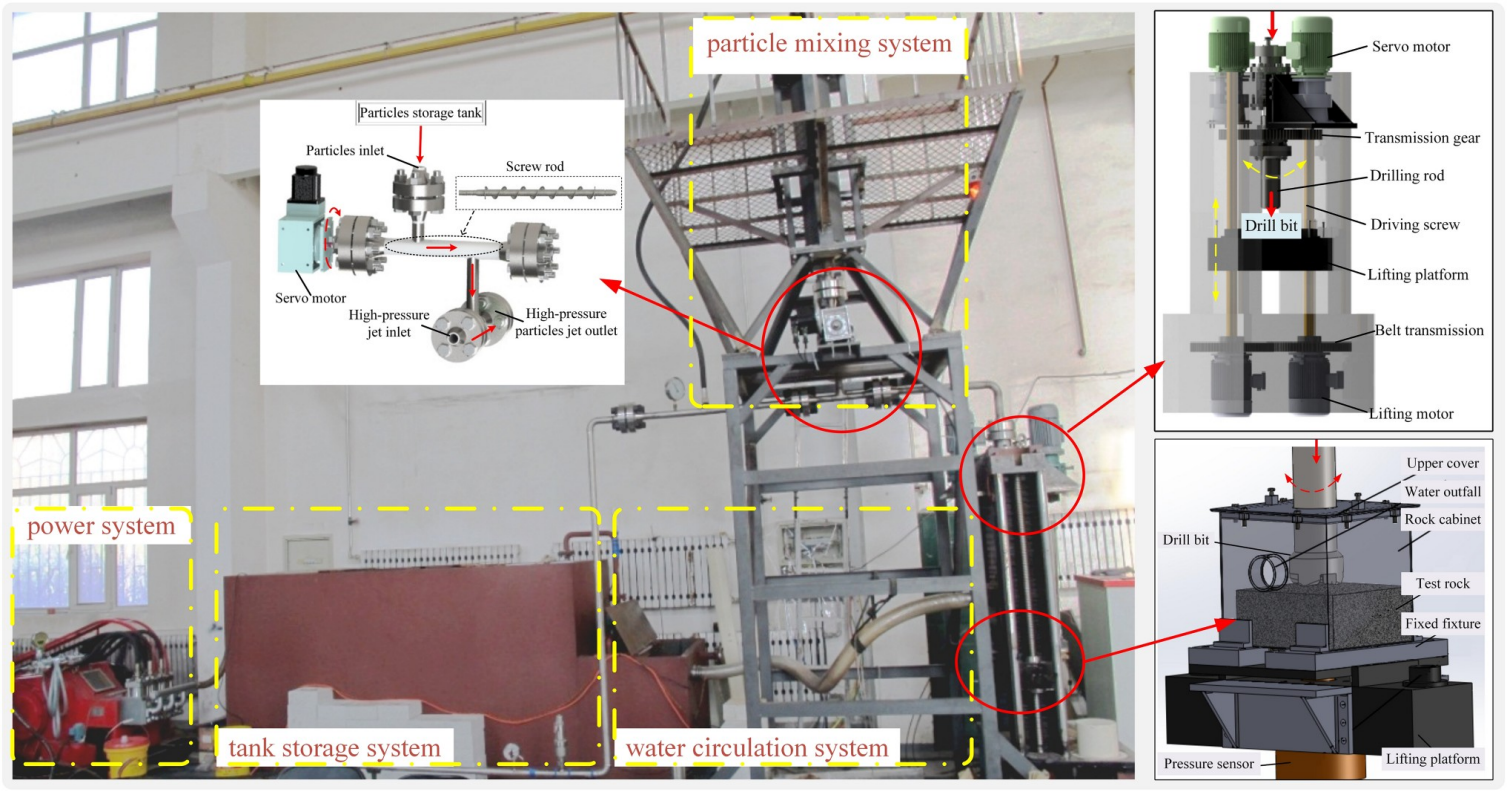
Particle jet coupled impact rock-breaking device in rotary drilling.

According to working principle and key technologies, a set of particle jet coupled impact rock-breaking device in rotary drilling was self-developed, as shown in Fig 11. The rock-breaking experimental materials mainly include steel particles, hard granite and clear water. Spherical particles are made of carbon steel [20]; clean water is instead of drilling fluid, and hard granite (Pingyi quarry, Shandong Province) is used for rock-breaking sample. The experimental parameters are shown in Table 3.

**Table 3:** The experimental parameters.

Pump speed (r/min)	Speed of simulated top-drive (r/min)	Speed of propeller (r/min)	Jet velocity (m/s)
80~180	0~100	0~40	0~280
Operating pressure (MPa)	Plunger diameter (mm)	plunger travel (mm)	Flow rate of waterjet (m^3^/h)
32	70	80	0~10.8

The merit of self-developed experiment equipment can be summarized as: (1) The simulated top drive is designed, which can realize the drilling pressure up to 0~70KN in rotary cutting state of bit; (2) It can realize the rock fracture under impact of particle waterjet with jet velocity of 0~280m/s, while the bit drills concurrently at a speed of 0~100r/min in rotary state. (3) It can realize the comprehensive rock-breaking experiments under impact of different objects, such as waterjet, particle and abrasive. (4) It can provide hydrodynamic power of 10.8m^3^/h and 32Mpa, and can realize rock-breaking effect under different impact velocity, particle size, angle and particle ratio.

**Fig 12:**
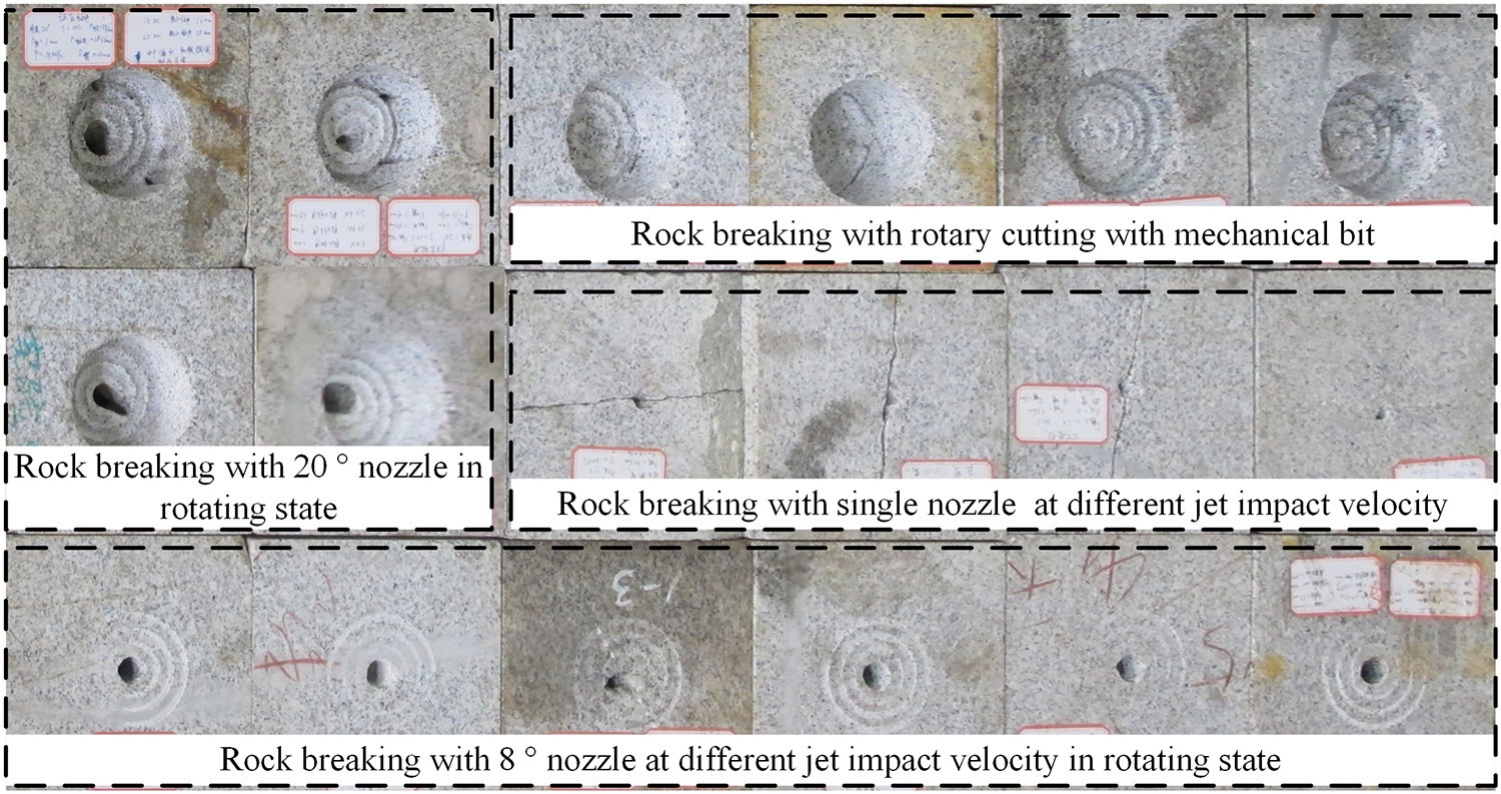
The rock broken samples and measurement method.

With coupled impact of particle waterjet, rock sample is broken for irregular hole and ring, and the rock-broken effect is shown in Fig 12. The measurement method of rock failure volume and penetration depth is “equivalent water injection method” and “laser ranging method”. The “equivalent water injection method” is to immerse the rock in water to make it in state of water saturation, and then inject water into the broken hole, and the water-injected volume is crush volume of rock. The “laser ranging method” refers to use laser as the light source for ranging. In process of measurement, repeated measurements are made to take the average value to reduce manual error.

### Result analysis and discussion

Based on the established numerical model and rock-breaking lab experiment results, the comparison and verification of penetration depth and failure volume under impact of particle waterjet are conducted to analyze the influence of jet parameters on rock-breaking effect and the similarities and differences between particle jet impact drilling and traditional drilling.

#### Comparative analysis with conventional drilling

**Fig 13:**
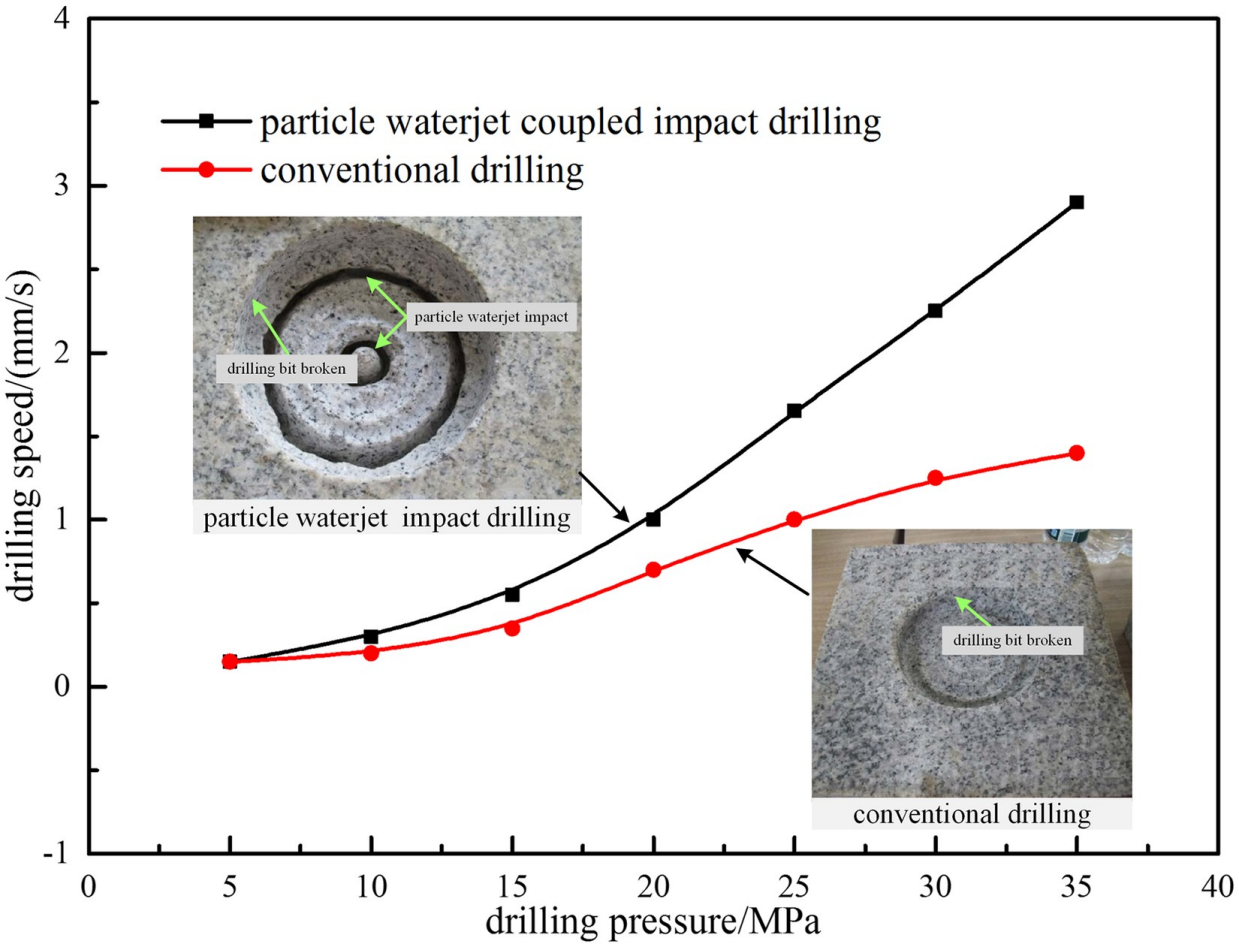
The drilling speed with different drilling pressure.

In deep-well hard formation drilling process, the constant-speed drilling technology is difficult to achieve in engineering, while the constant-pressure drilling technology is easier to achieve and operate. As long as the cutting speed of bit teeth is less than that of particle waterjet, particle jet coupled impact drilling technology can be satisfied the drilling process. Therefore, the comparative experiment of particle jet coupled impact and drilling bit cutting is carried out. During experiment, the mode of particle waterjet combined bit is adopted, while for conventional drilling, only bit is used to break rock. The experimental results with different drilling pressure are shown in Fig 13.

According to rock-breaking samples with particle waterjet impact and drilling bit cutting, the impact of particle waterjet mainly forms annular pressure-relief groove, and the rotary cutting of drilling bit mainly forms borehole. The drilling speed curve shows that the difference of drilling speed between conventional drilling and particle jet coupled impact drilling at low drilling pressure is not obvious; when drilling pressure continues to rise, the difference would be gradually obvious, and when pressure is 35KN, the difference of drilling speed is more than one time. The reasons is that: when drilling pressure is low, the pressure can’t reach rock failure strength, so the difference of drilling speed is not significant; with increasing of drilling pressure, a ring groove is broken on the bottom rock surface in advance by particle waterjet, the compaction effect is eliminated and rock failure strength is reduced, so drilling speed could be significantly increased. Therefore, the advantages of particle waterjet impact drilling technology can be highlighted to a greater extent when it is used to break the hard strata in deep-wells.

In addition, due to the complexity of rock-breaking, these parameters can also be adjusted by real-time monitoring of drilling pressure and the footage rate. When the footage speed is fast and the drilling pressure is small, particle ratio and jet velocity can be reduced properly; when particle ratio and jet velocity cannot be increased significantly, the drilling pressure can be increased properly to obtain the fast footage speed.

#### Effect of impact angle on rock-breaking

**Fig 14:**
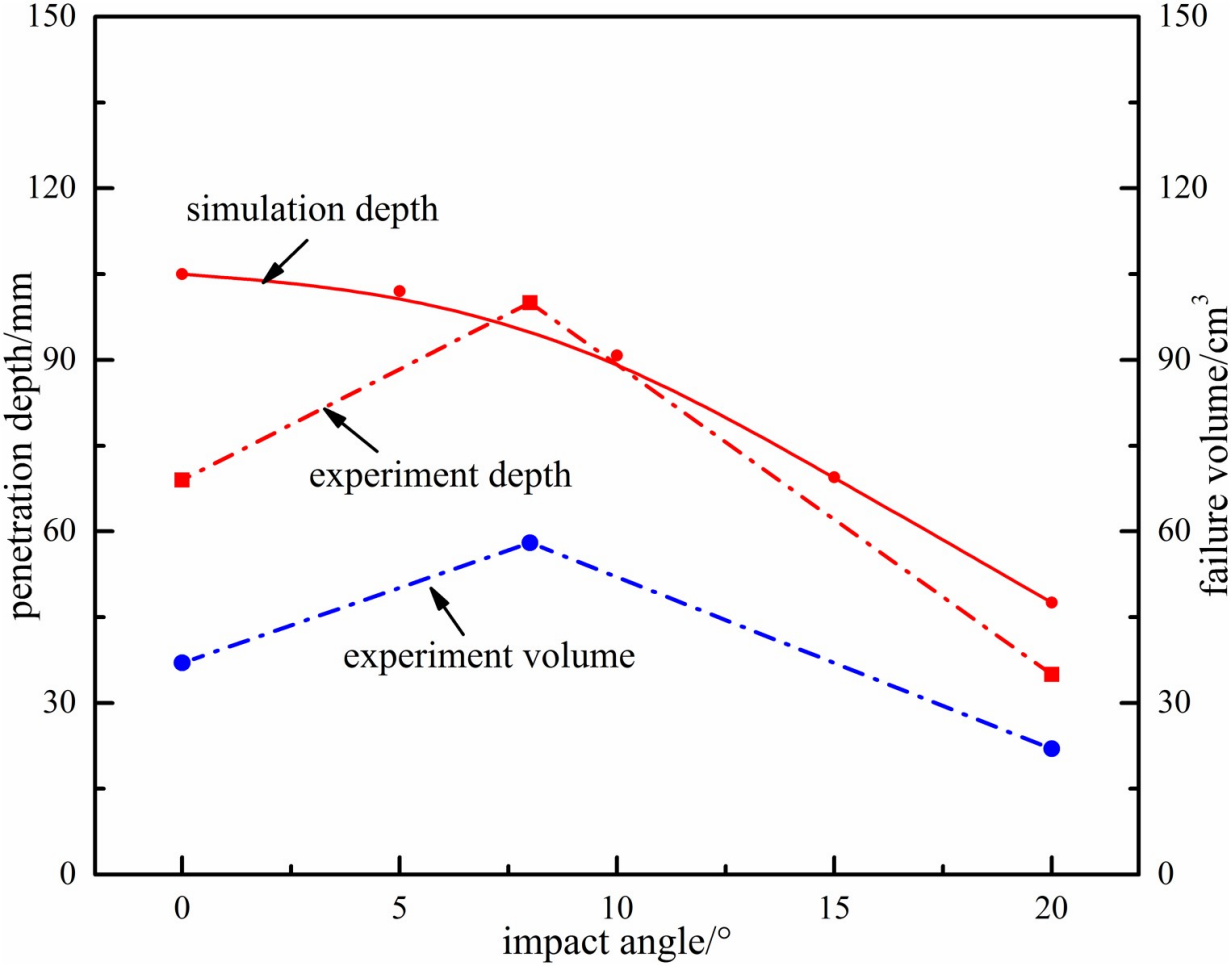
The effect of impact angle on failure volume and penetration depth.

The impact angle refers to the angle between the axis of water jet and the axis of bit. The rock breaking effect is obviously different under different impact angles. Based on the particle jet impact rock breaking experiment, the rock breaking effect is analyzed when the impact angle is 0°, 8° and 20°, and penetration depth under different impact angles was studied with numerical simulation. The experiment and simulation results with different angles are shown in Fig 14 (experiment time is 50s, particle size is 1.0mm, and jet velocity is 200m/s).

According to the results analysis, with increase of impact angle, penetration depth and failure volume would increase first and then decrease. And the maximum penetration depth is about 3~4 times of the minimum depth. Quantitative analysis shows, there is a critical value of impact angle (0°~10°), which can not only reach the maximum value of depth, but also achieve the purpose of removing rock debris. Because there would be collision and interference when impact angle is 0°, and also there would be more energy loss because the angle is too large. In process of engineering drilling, the impact angle and number of nozzles should be reasonably arranged according to experimental results, and it is suggested to use the combination layout of a nozzle with 8° and three or four nozzles with 20° for rapidly rock-breaking.

Because rock-breaking experiment volume contains the crushing effect of bit, the simulated volume is not verified. The penetration depth in simulation state is basically consistent with that in experiment state, which prove the correctness and applicability of simulation model.

#### Effect of jet velocity on rock-breaking

**Fig 15:**
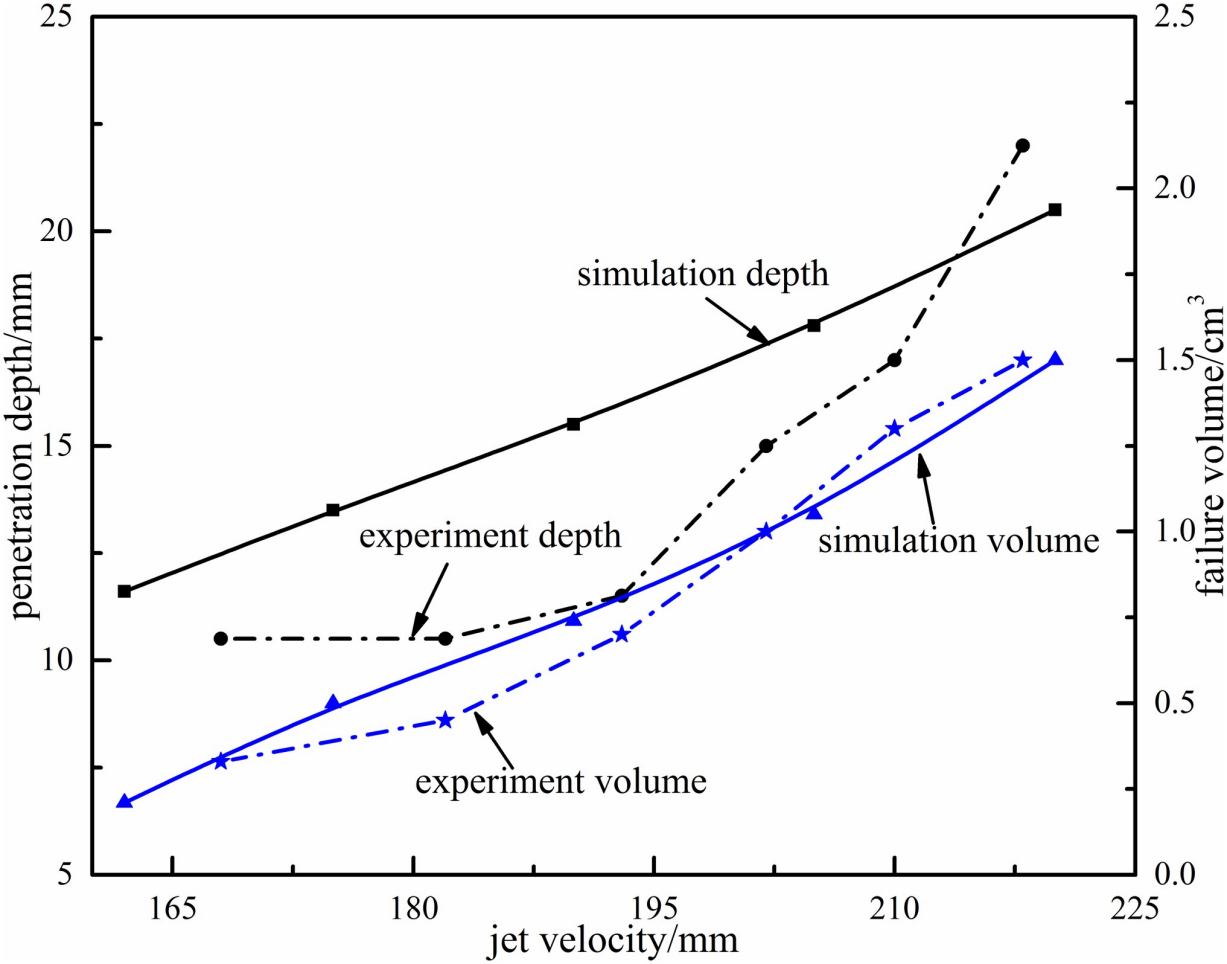
The effect of jet velocity on failure volume and penetration depth.

The jet velocity determines impact kinetic energy and acceleration ability, so jet velocity plays an important role in rock-breaking process. In order to analyze the effect of jet velocity on failure volume and penetration depth, the rock-breaking experiment under particle waterjet coupled impact is carried out, and compared with dynamic simulation. The experimental and simulation results with different velocities are shown in Fig 15 (experiment time is 10s, impact angle is 8°, and particle size is 1.0mm).

According to the analysis of experimental results, when jet velocity is low, the penetration depth and failure volume are small. And when jet velocity exceeds 180m/s, failure volume and penetration depth would increase rapidly. Therefore, the higher the jet velocity, the greater the penetration depth and failure volume. Comparing the simulation results with experiment results, the change trend of penetration depth and failure volume is basically consistent in qualitative analysis, and the average error is within 5% in quantitative analysis. The research results prove the correctness and applicability of JH-2 model in particle impact rock-breaking simulation.

## Conclusion

In this paper, the rock-breaking performance of particle jet coupled impact drilling technology is analyzed by studying penetration depth and failure volume with comparison of lab experiment and numerical simulation, and the main researches are as follows:

1. The working principle of particle jet impact rock-breaking in rotary drilling was introduced, and the acceleration mechanism of particle waterjet is studied, which shows that waterjet velocity can be accelerated to about 13 times, and particle velocity can be accelerated to about 11 times. And damage evolution and broken effect at such high-velocity impact is simulated, which reveals that damage evolution under particle jet impact presents radiating expansion, and rock-breaking effect will be better when particle velocity exceeds 150m/s.
2. Particle jet coupled impact experimental equipment in rotary drilling is self-developed, and the penetration depth and failure volume experiments were comparative verified with numerical simulation. Result showed that combination nozzles layout of impact angle with 8°and 20° are suggested to adopt to achieve rock-drilled rapidly, which also demonstrated the correctness and applicability of simulation model.
3. Comparative analysis with conventional drilling, drilling speed with particle jet impact is twice that of conventional drilling, which demonstrates the advantages and engineering application significance of particle jet coupled impact rock-breaking technology in rotary drilling.

## Supporting information

S1 File (ZIP)

S1 Table The parameters of particle and waterjet. (DOC)

S2 Table The model parameters of rock damage simulation. (DOC)

S3 Table The experimental parameters. (DOC)
