# Supplementary figures and images for: Particle jet impact deep-rock in rotary drilling: Failure process and lab experiment
Source: PLoS One. 2021 Apr 28;16(4):e0250588. doi: 10.1371/journal.pone.0250588 (PMC8081264; doi:10.1371/journal.pone.0250588)

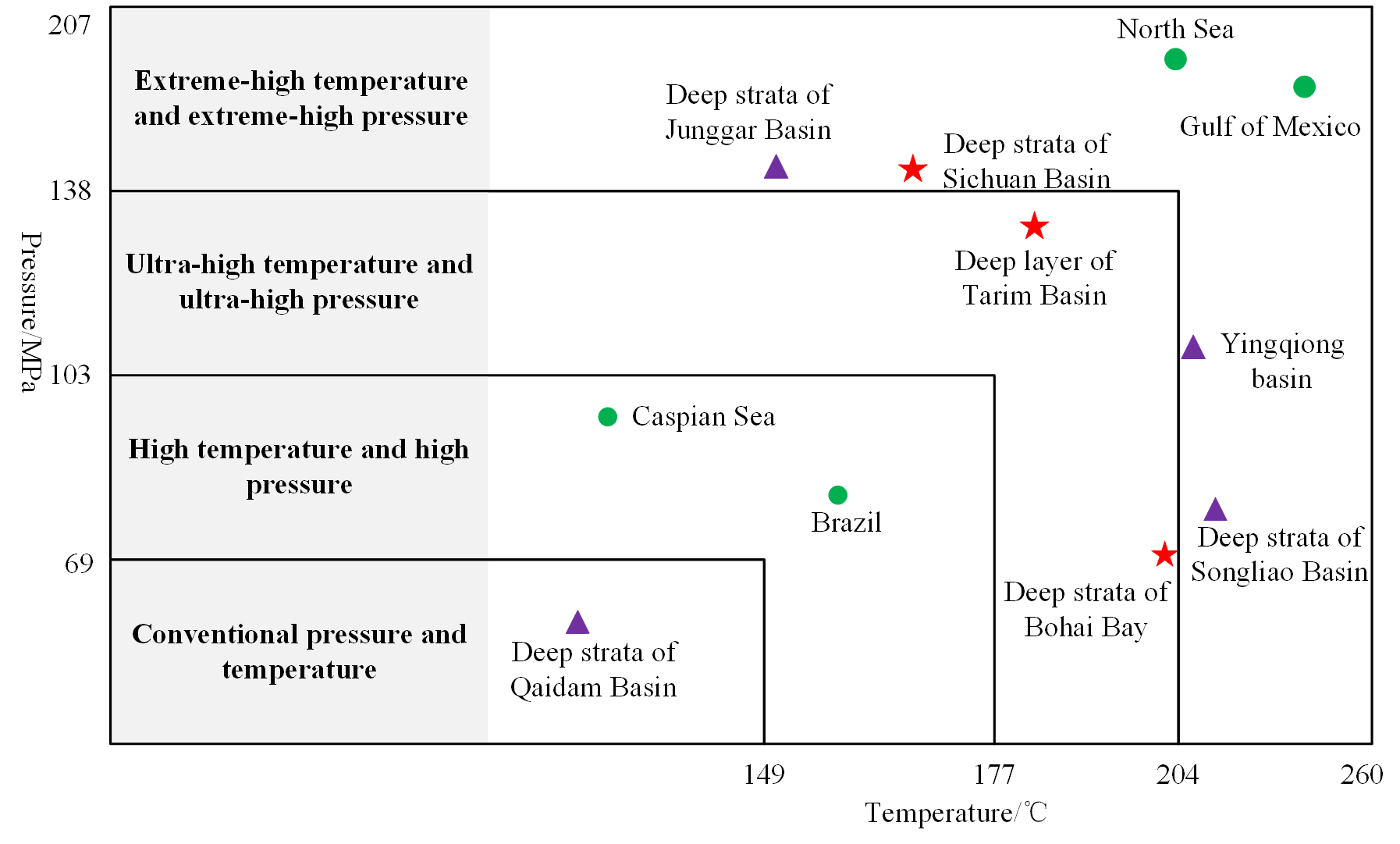

Supplement: S1 File — (ZIP) [file pone.0250588.s001.zip › S1 Fig/Fig 1.tif]

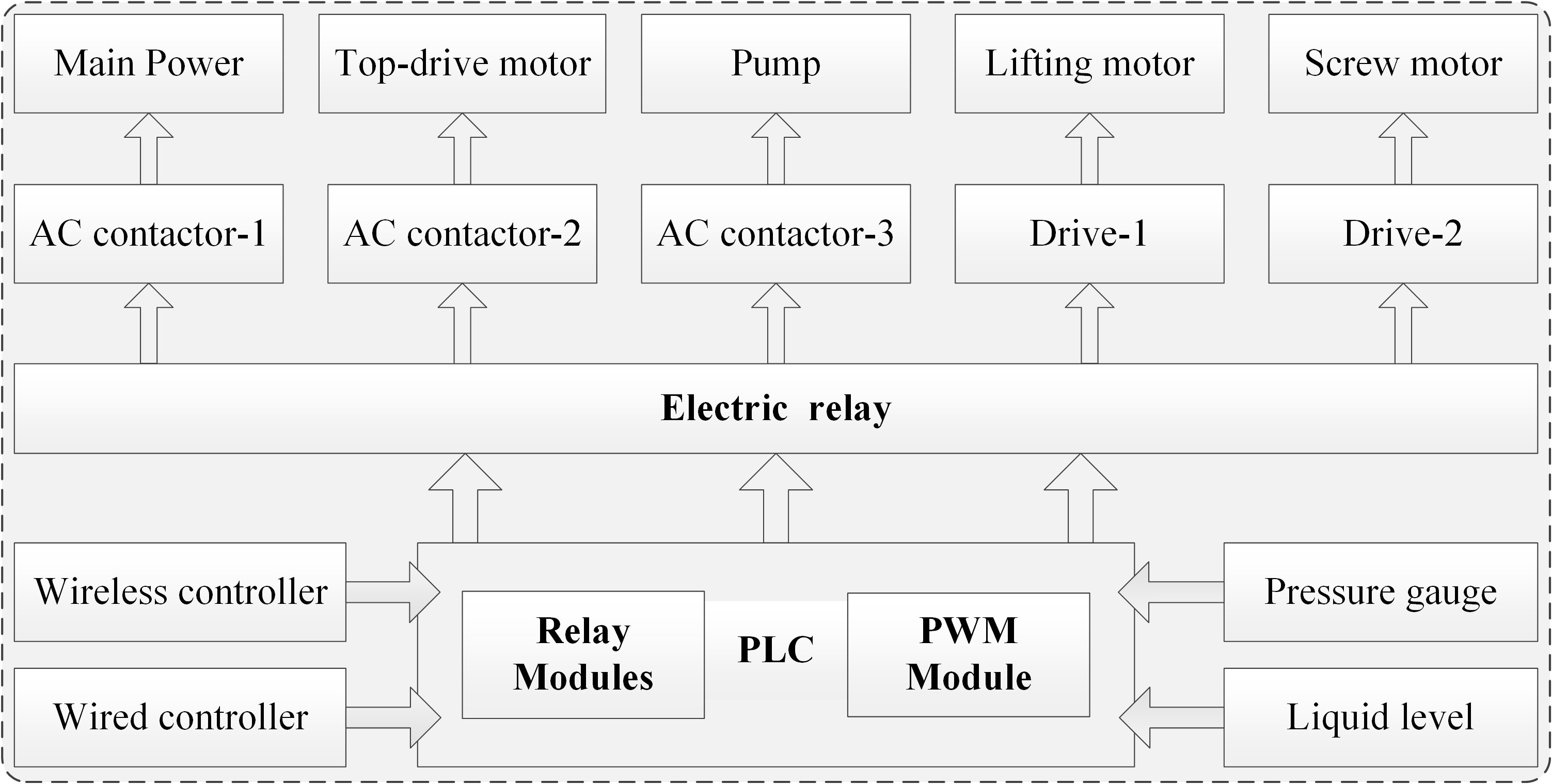

Supplement: S1 File — (ZIP) [file pone.0250588.s001.zip › S1 Fig/Fig 10.tif]

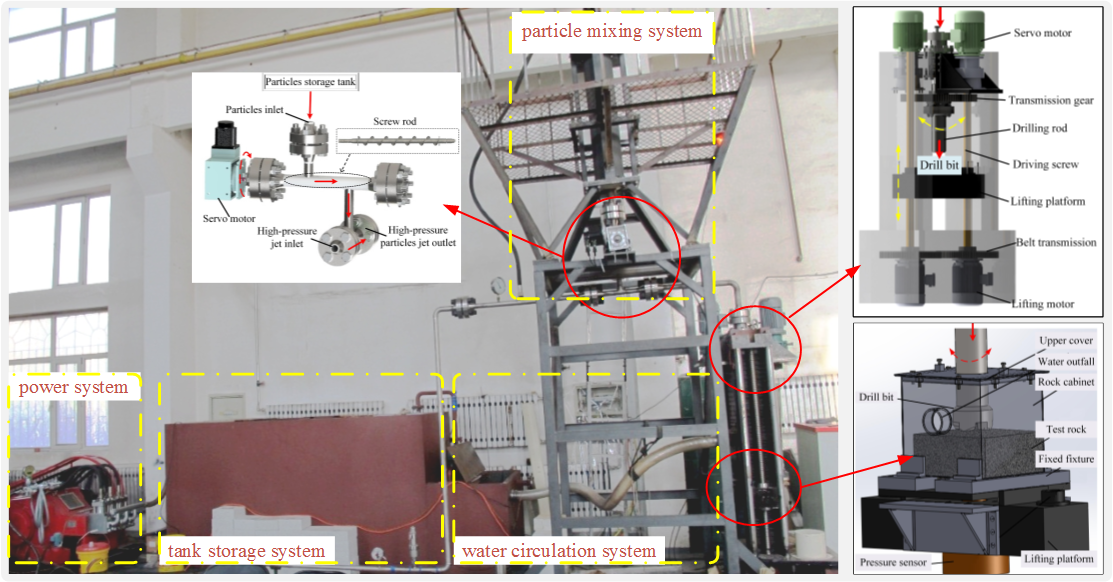

Supplement: S1 File — (ZIP) [file pone.0250588.s001.zip › S1 Fig/Fig 11.tif]

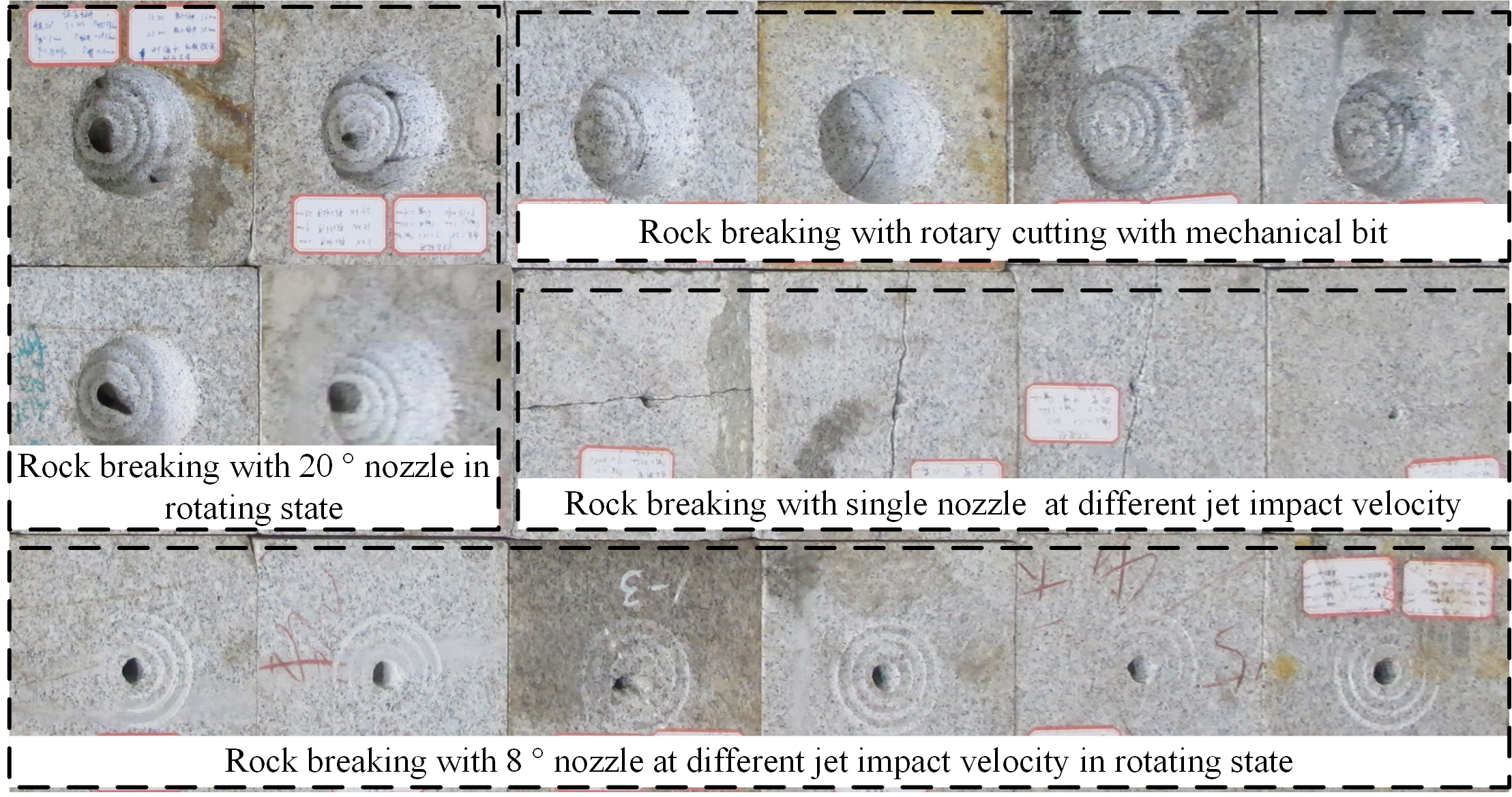

Supplement: S1 File — (ZIP) [file pone.0250588.s001.zip › S1 Fig/Fig 12.tif]

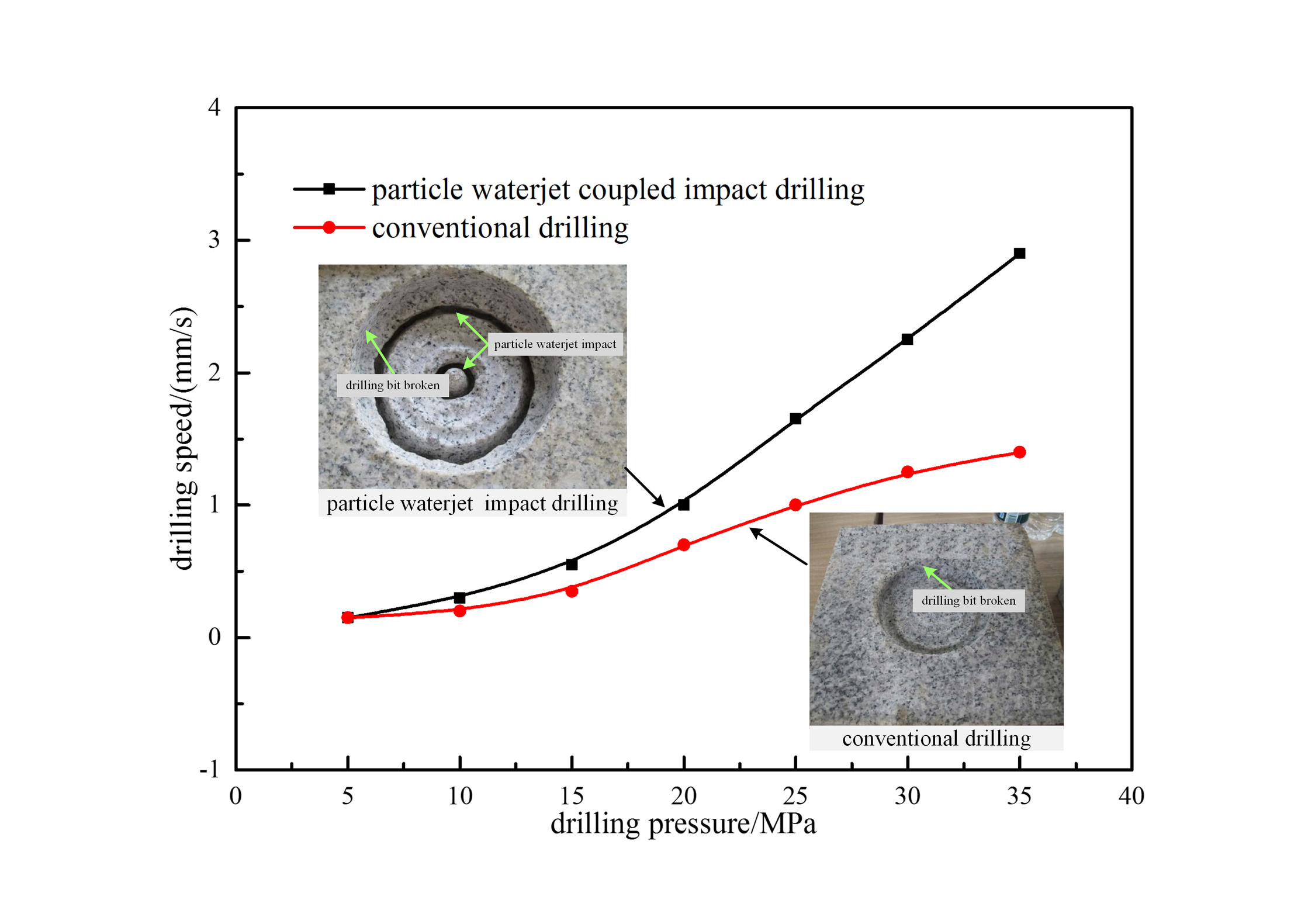

Supplement: S1 File — (ZIP) [file pone.0250588.s001.zip › S1 Fig/Fig 13.tif]

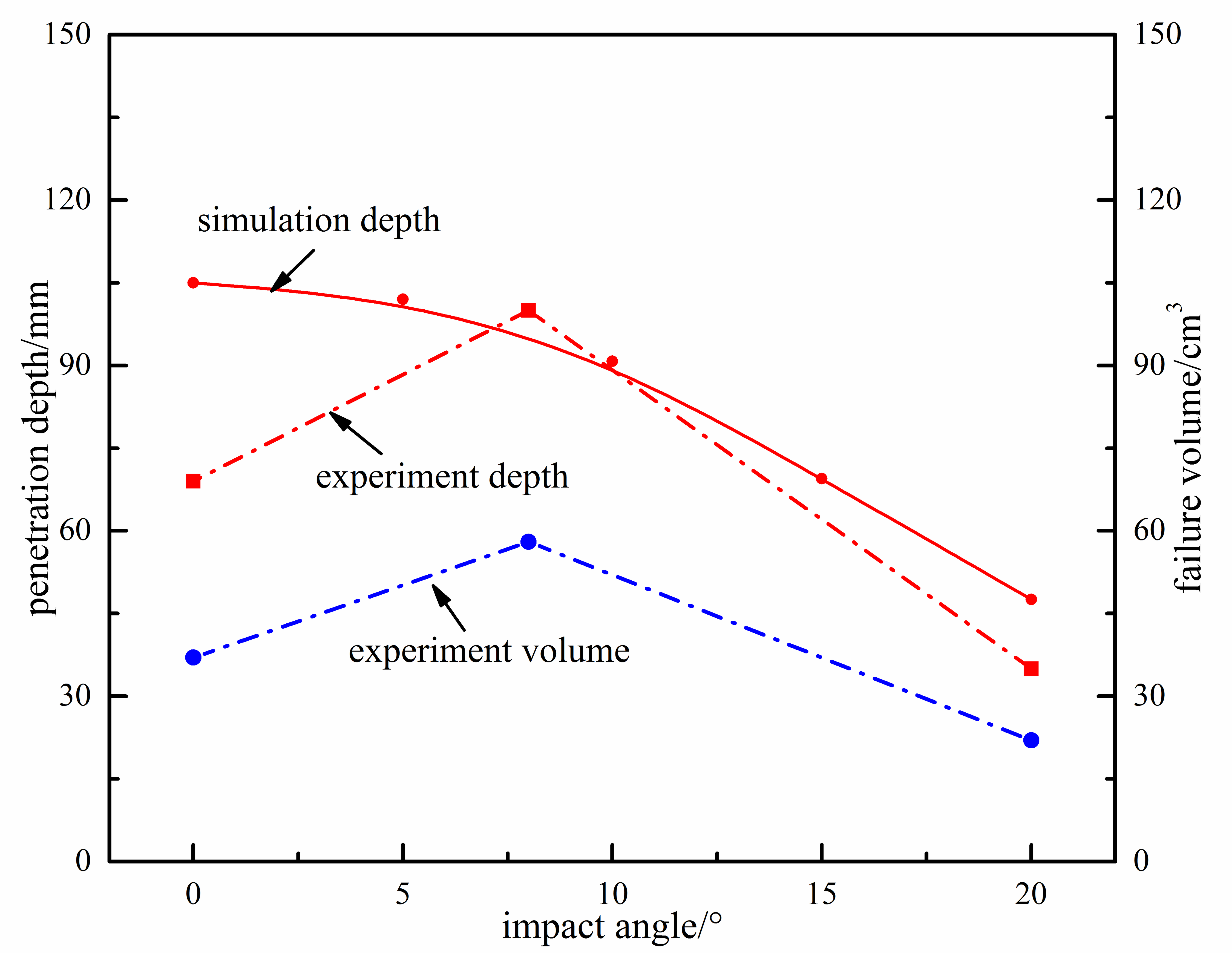

Supplement: S1 File — (ZIP) [file pone.0250588.s001.zip › S1 Fig/Fig 14.tif]

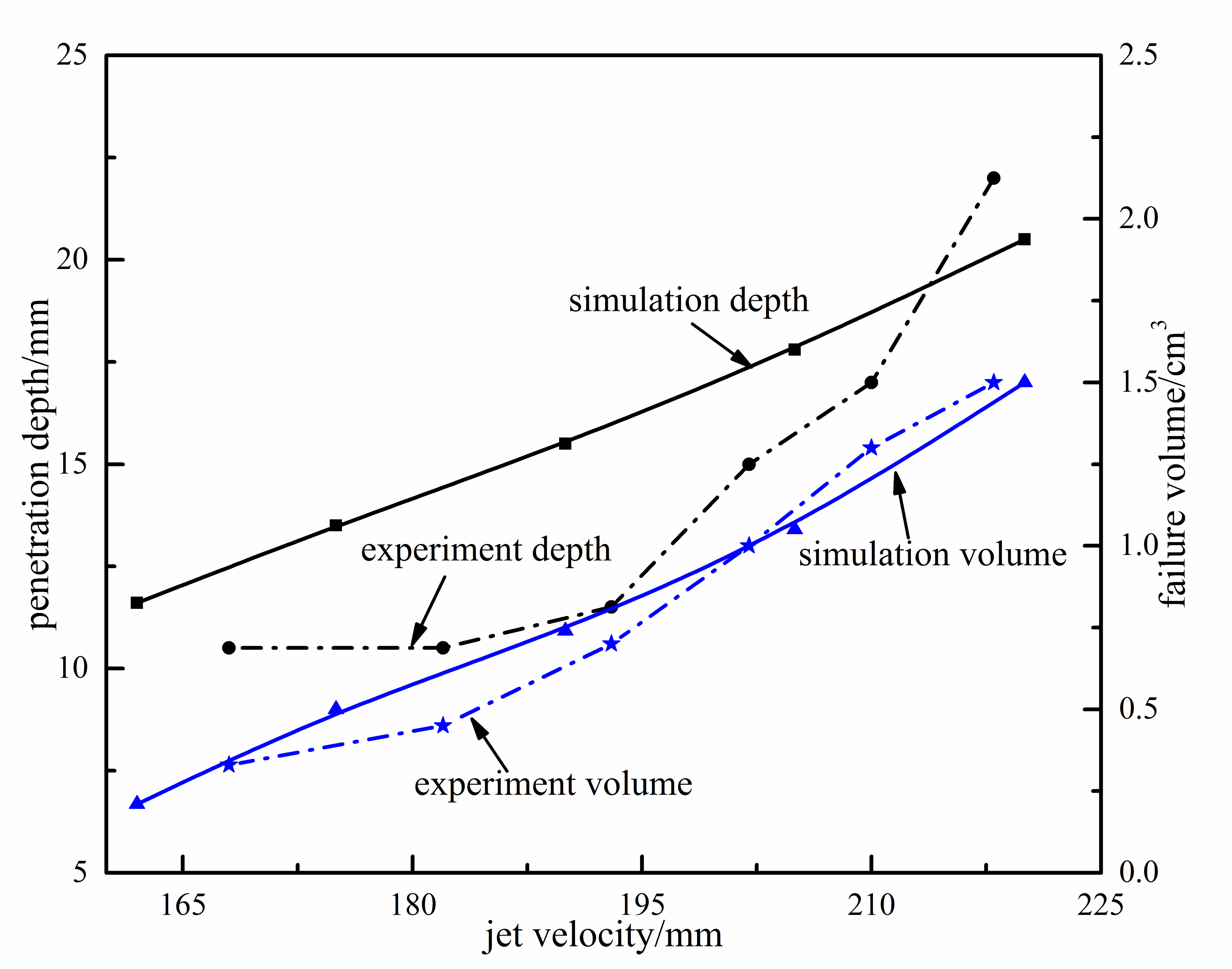

Supplement: S1 File — (ZIP) [file pone.0250588.s001.zip › S1 Fig/Fig 15.tif]

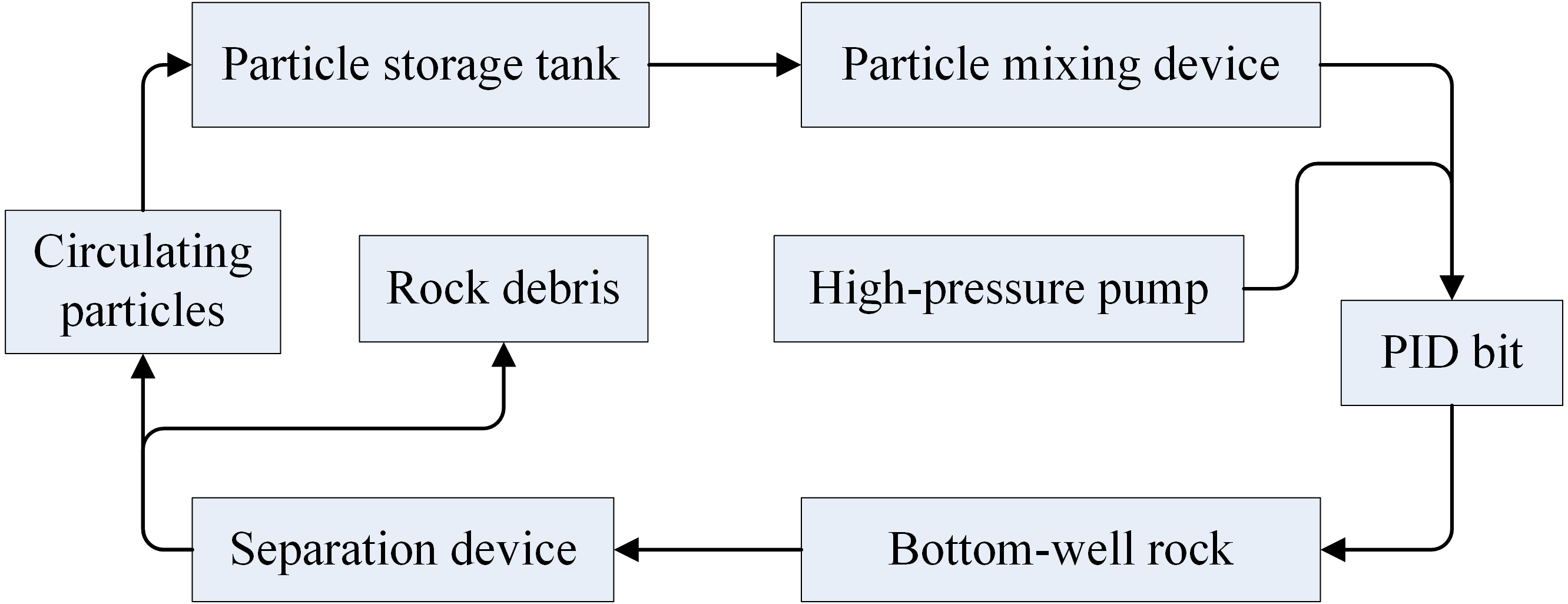

Supplement: S1 File — (ZIP) [file pone.0250588.s001.zip › S1 Fig/Fig 2.tif]

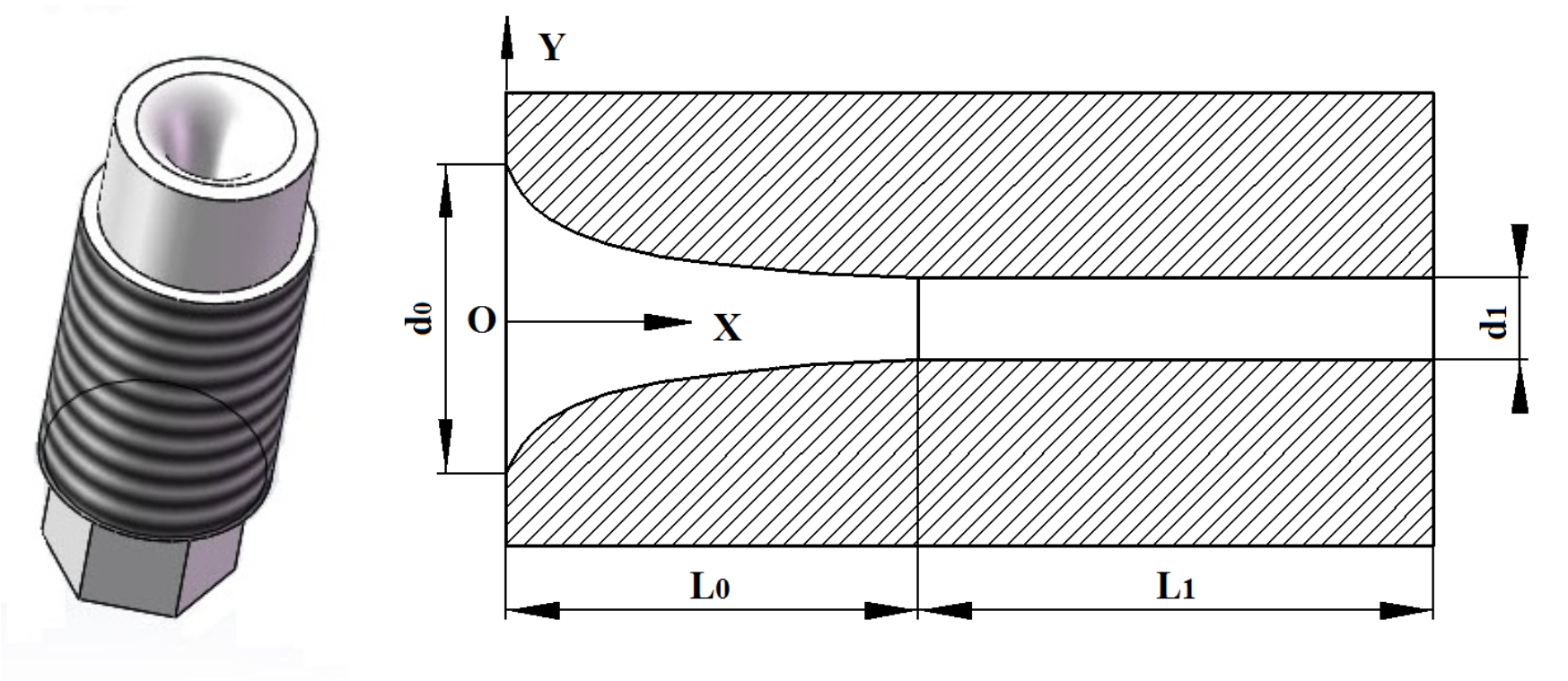

Supplement: S1 File — (ZIP) [file pone.0250588.s001.zip › S1 Fig/Fig 3.tif]

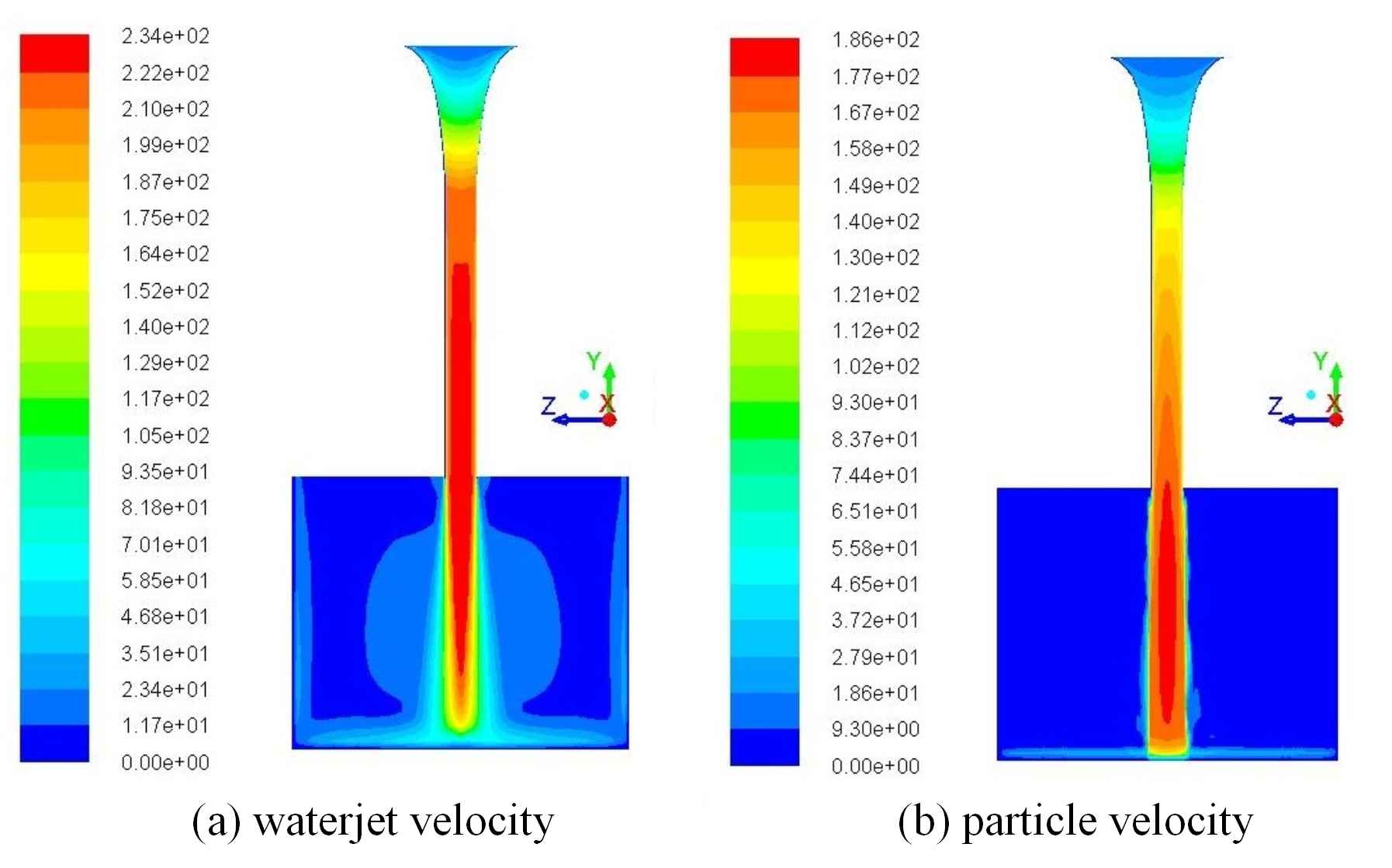

Supplement: S1 File — (ZIP) [file pone.0250588.s001.zip › S1 Fig/Fig 4.tif]

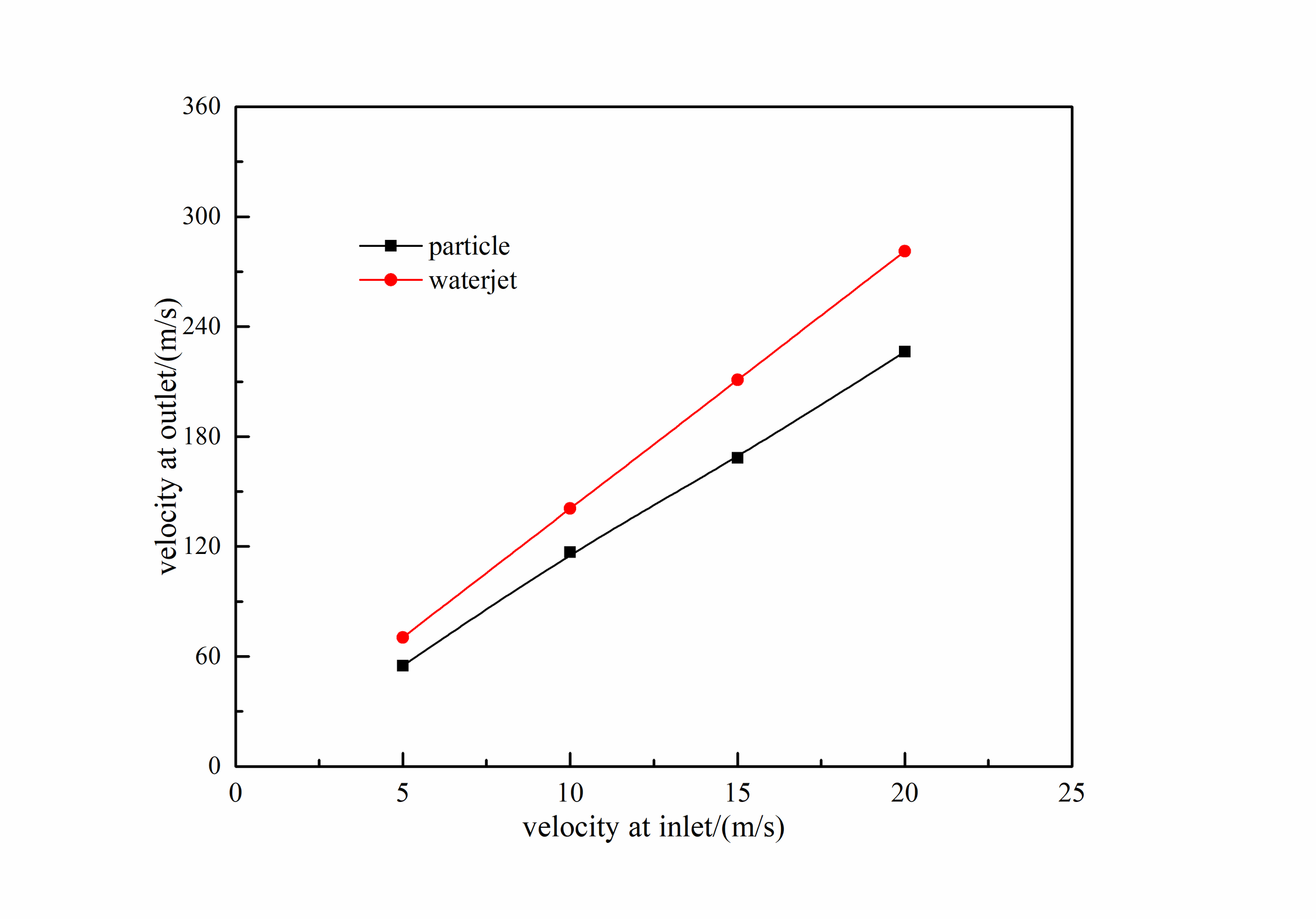

Supplement: S1 File — (ZIP) [file pone.0250588.s001.zip › S1 Fig/Fig 5.tif]

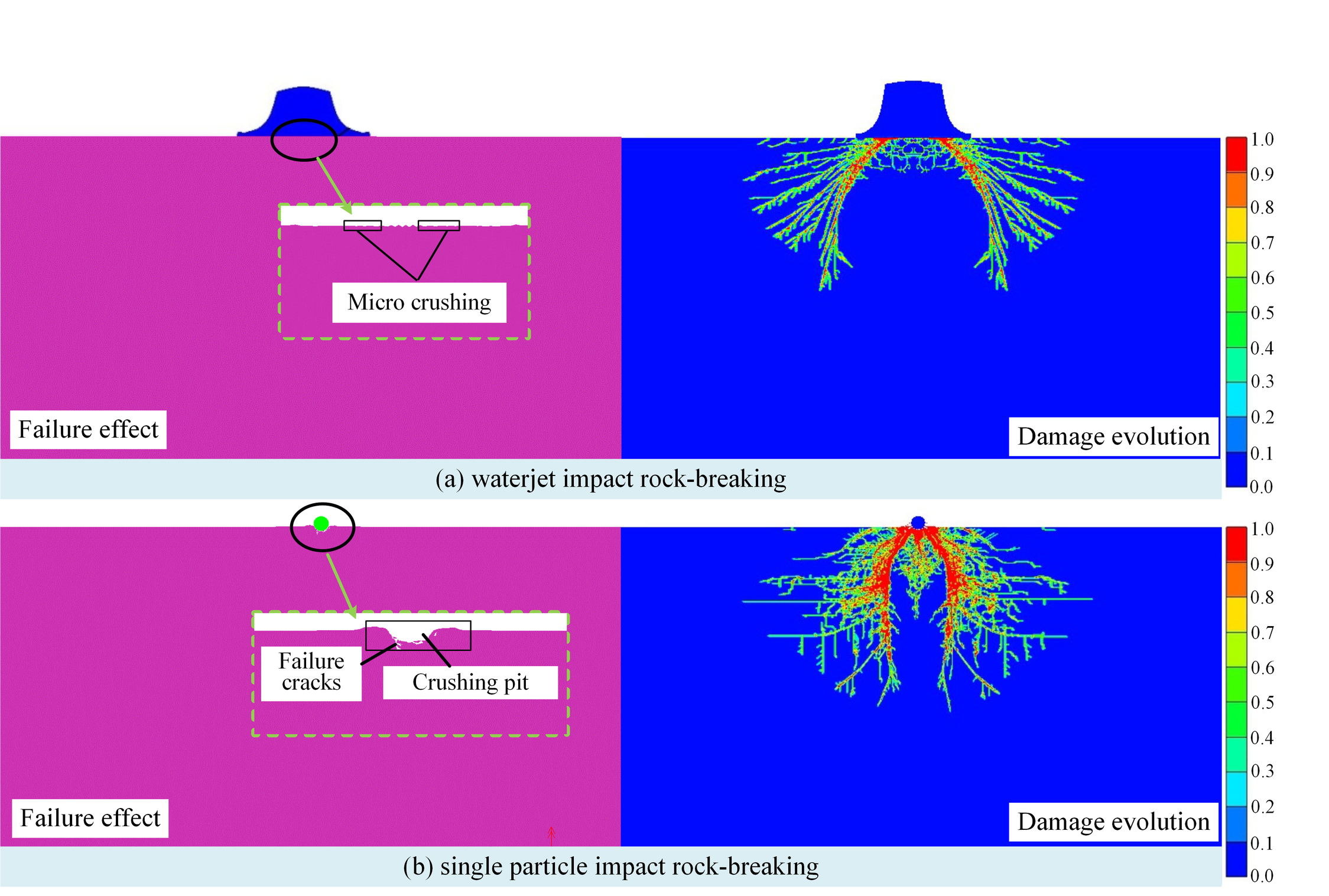

Supplement: S1 File — (ZIP) [file pone.0250588.s001.zip › S1 Fig/Fig 6.tif]

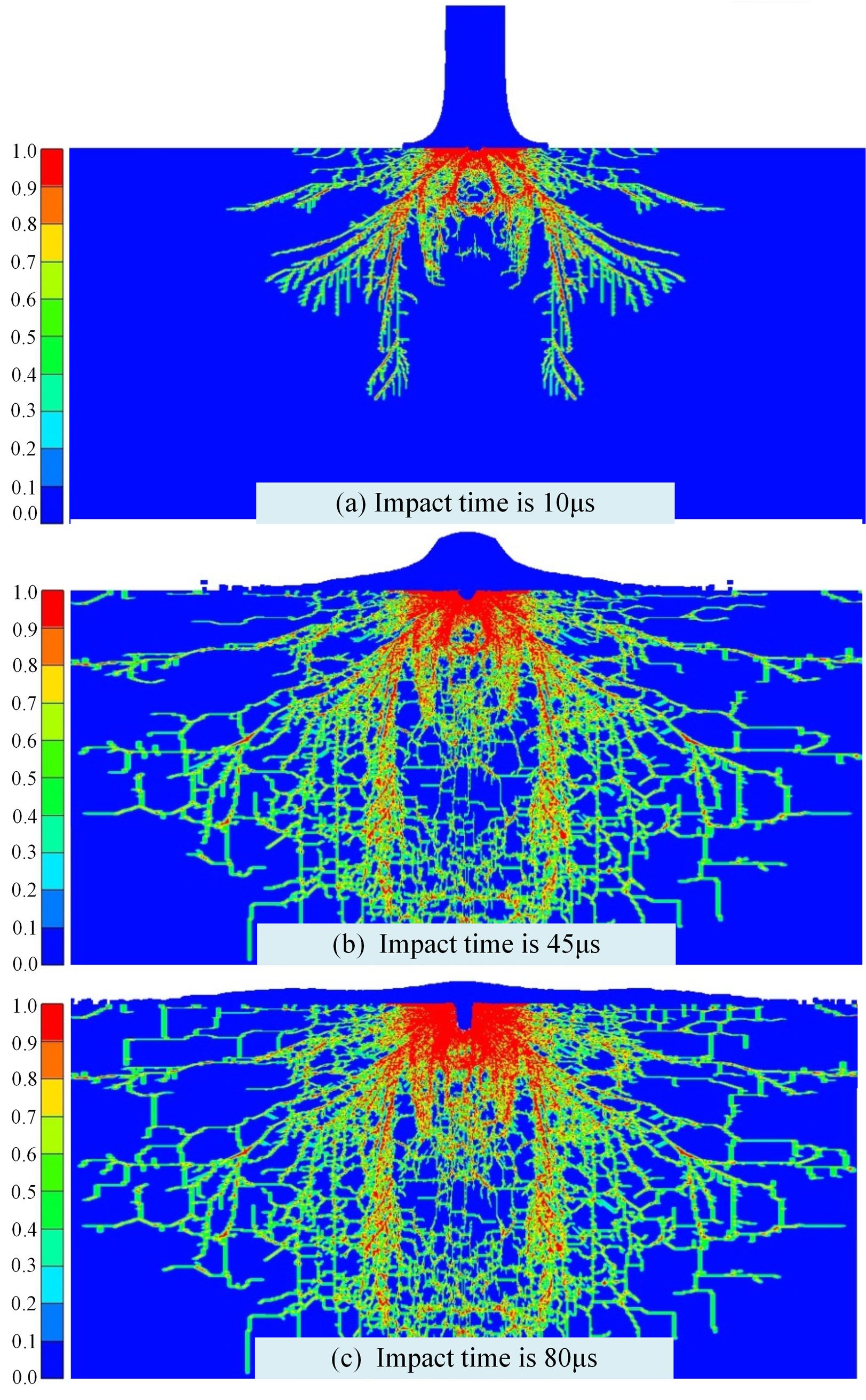

Supplement: S1 File — (ZIP) [file pone.0250588.s001.zip › S1 Fig/Fig 7.tif]

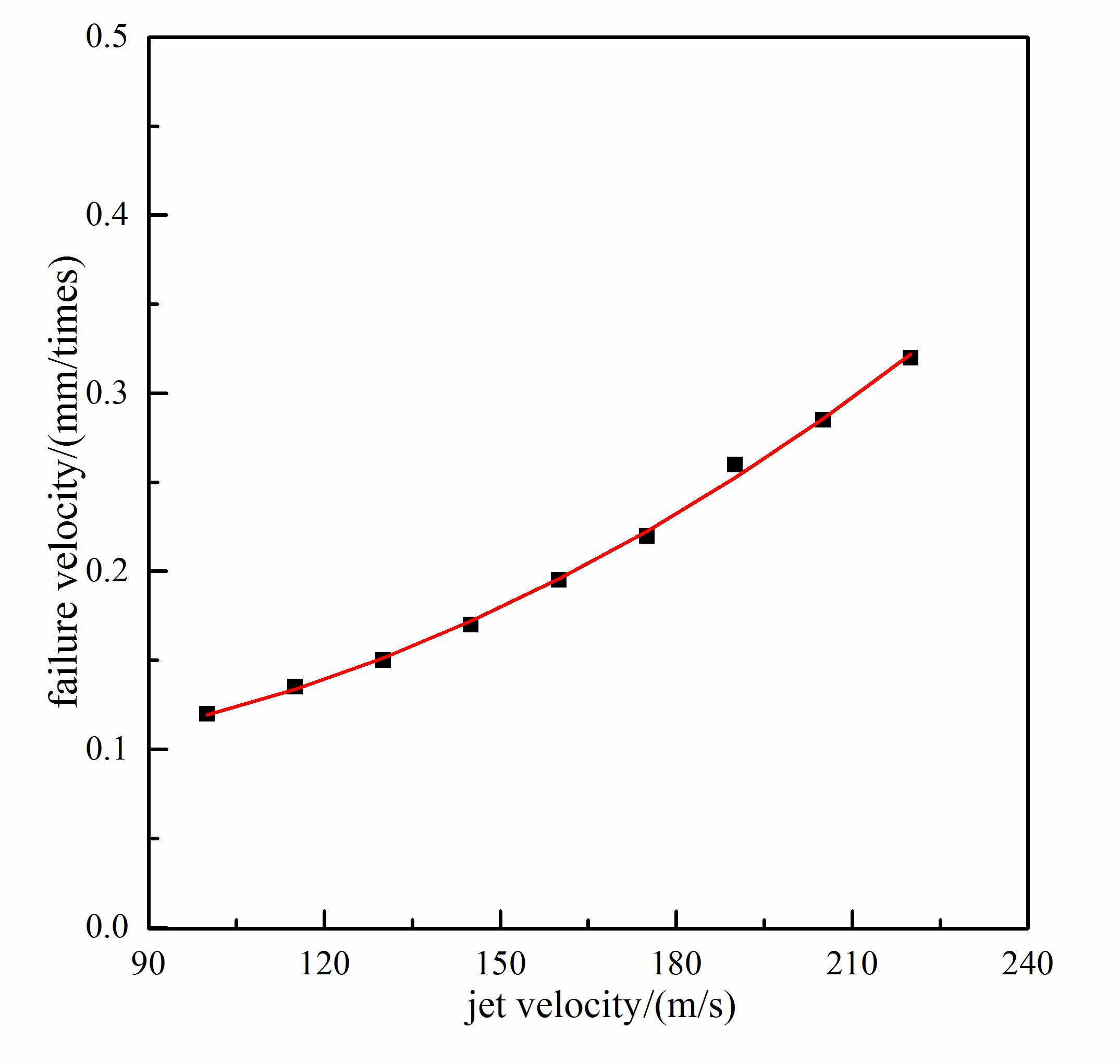

Supplement: S1 File — (ZIP) [file pone.0250588.s001.zip › S1 Fig/Fig 8.tif]

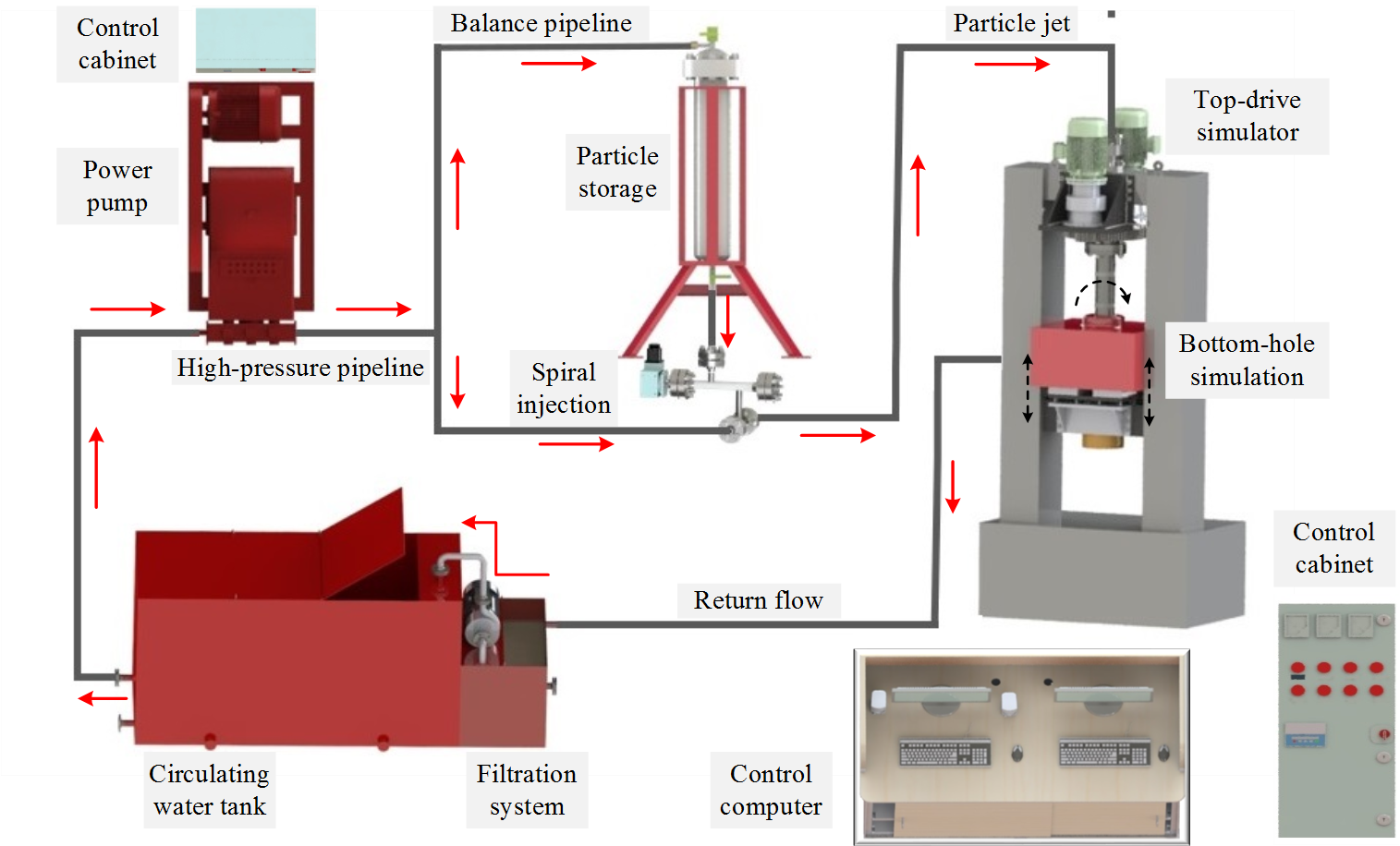

Supplement: S1 File — (ZIP) [file pone.0250588.s001.zip › S1 Fig/Fig 9.tif]
