# Supplementary material for: Particle jet impact deep-rock in rotary drilling: Failure process and lab experiment
Source: PLoS One. 2021 Apr 28;16(4):e0250588. doi: 10.1371/journal.pone.0250588 (PMC8081264; doi:10.1371/journal.pone.0250588)
Supplement: S1 Table — (DOC) [file pone.0250588.s002.doc]

**S1 Table.** The parameters of particle and waterjet

| jet density$\rho_{f}$ | Particle density$\rho_{p}$ | jet velocity at inlet$v_{f0}$ | Particle diameter$D_{P}$ | Viscosity coefficient$C_{D}$ |
| --- | --- | --- | --- | --- |
| 1100 kg/m^3^ | 7800 kg/m^3^ | 5~20 m/s | 1.0 mm | 17.2 μPa·s |
| Diameter of inlet$d_{0}$ | Diameter of outlet$d_{1}$ | Contraction length$L_{0}$ | Cylinder length$L_{1}$ | Compressibility of fluid$Y_{M}$ |
| 15 mm | 4 mm | 15 mm | 40 mm | 0 |
