# Supplementary material for: Particle jet impact deep-rock in rotary drilling: Failure process and lab experiment
Source: PLoS One. 2021 Apr 28;16(4):e0250588. doi: 10.1371/journal.pone.0250588 (PMC8081264; doi:10.1371/journal.pone.0250588)
Supplement: S2 Table — (DOC) [file pone.0250588.s003.doc]

**S2 Table.** The model parameters of rock damage simulation

| Rock density$\rho_{r}$ | Bulk modulus$K_{1}$ | Shear modulus$G$ | Poisson's ratio$\mu$ |
| --- | --- | --- | --- |
| 2600 kg/m^3^ | 25.6 GPa | 20.4 GPa | 0.30 |
| Damage constant$D_{1}$ | Damage constant$D_{2}$ | Compressive strength$F_{c}$ | Effective stress of elastic limit$\sigma_{Hel}$ |
| 0.05 | 0.95 | 55 MPa | 4.52 GPa |
