# Supplementary material for: Particle jet impact deep-rock in rotary drilling: Failure process and lab experiment
Source: PLoS One. 2021 Apr 28;16(4):e0250588. doi: 10.1371/journal.pone.0250588 (PMC8081264; doi:10.1371/journal.pone.0250588)
Supplement: S3 Table — (DOC) [file pone.0250588.s004.doc]

**S3 Table.** The experimental parameters

| Pump speed (r/min) | Speed of simulated top-drive (r/min) | Speed of propeller (r/min) | Jet velocity (m/s) |
| --- | --- | --- | --- |
| 80~180 | 0~100 | 0~40 | 0~280 |
| Operating pressure (MPa) | Plunger diameter (mm) | plunger travel (mm) | Flow rate of waterjet (m^3^/h) |
| 32 | 70 | 80 | 0~10.8 |
